# Lifecycle Implications of Poly(vinyl chloride) (PVC) Micro(nano)plastics (MNPs): Interactions with Coexposed Environmental Pollutants (EPs) and Impact on Their Toxicity and Bioavailability

**DOI:** 10.1021/acs.est.5c14500

**Published:** 2026-05-14

**Authors:** Satwik Majumder, Glen DeLoid, Milton Das, Mandeep Kaur, Eshun Gaddi, Sarah Alotaibi, Nubia Zuverza-Mena, Omowunmi Sadik, Jason White, Philip Demokritou

**Affiliations:** † Nanoscience and Advanced Materials Center, Environmental and Occupational Health Sciences Institute (EOHSI), Rutgers Biomedical Health Sciences, 151434 Rutgers University, Piscataway, New Jersey 08854, United States; ‡ Department of Analytical Chemistry, The Connecticut Agricultural Experiment Station, New Haven, Connecticut 06511, United States; § Department of Chemistry and Environmental Sciences 161 Warren Street, New Jersey Institute of Technology, University Heights, Newark, New Jersey 07102, United States

**Keywords:** micro(nano)plastics (MNPs), environmental pollutants (EPs), small intestinal epithelium (SIE), poly(vinyl chloride) (PVC), mechanical fragmentation, photo-oxidation, plastic incineration, bioavailability

## Abstract

Sorption of environmental pollutants (EPs) on micro­(nano)­plastics (MNPs) across their lifecycle raises significant concerns regarding potential toxicological effects, but remains understudied. To address these knowledge gaps, we assessed the sorption of EPs, including toxic elements (arsenic (As), chromium (Cr), and lead­(Pb)), and organic pollutants (boscalid and PFOS) on poly­(vinyl chloride) (PVC) MNPs generated across their lifecycle by mechanical fragmentation/cryomilling, photo-oxidation, and incineration. The toxicological effects of EP-MNP coexposure were assessed using a small intestinal epithelium (SIE) model, coupled with a three-phase simulated GI digestion. Sorption of most EPs was MNP lifecycle-stage- and EP-specific, with aged-MNPs (cryomilled and UV photo-oxidized) having a higher EP affinity than unaged MNPs (cryomilled) and incinerated MNPs. While MNPs and EPs alone or combined did not produce cytotoxicity in the SIE, aged-MNPs and incinerated MNPs in the presence of EPs triggered oxidative stress, suggesting MNP lifecycle-stage and EP dependency. Importantly, MNP- and EP translocation across SIE were MNP concentration, MNP lifecycle-stage, and EP-dependent. Aged PVC (at 50 μg/mL) enhanced the translocation of Cr (78.8%), Pb (54.2%), boscalid (23.4%), and PFOS (56.9%), while incinerated PVC increased translocation of only boscalid (23.4%) and PFOS (56.9%) significantly when compared to EPs alone. EPs enhanced the translocation of aged PVC and incinerated PVC at 50 μg/mL by 102.2% and 54.0%, respectively, when compared to MNPs alone. Such reciprocal enhancement of the translocation of EPs and aged-MNPs, and of incinerated MNPs, correlated with the downregulation of cell junction genes, suggesting compromised cell junction integrity. Collectively, our findings offer significant toxicological insights into the lifecycle of ecologically relevant PVC MNPs, their interactions with EPs, and the consequent implications for the integrity of human intestinal epithelial structures.

## Graphical abstract

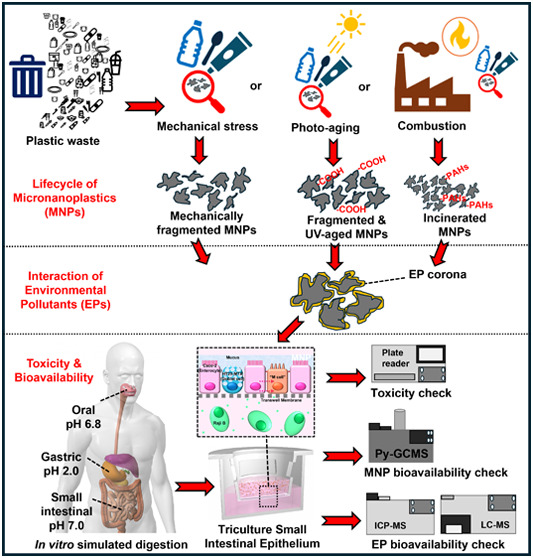

## 1 Introduction

The exponential increase in plastic production and the resultant accumulation of plastic debris in the environment present significant challenges for ecological integrity and public health.^1^ Since 1950, global plastic production has surged from 2 million metric tons to an estimated 400 million metric tons in 2022.^2^ Currently, the recycling rate for plastics remains critically low at just 9%, with an additional 12% being incinerated.^3^ Consequently, the majority of this waste is deposited directly into terrestrial and aquatic environments.^3^ Over time, plastic waste undergoes mechanical, thermal, and photo-oxidative breakdown, which culminates in the formation of micro­(nano)­plastics (MNPs).^1,3–9^ MNPs have emerged as persistent contaminants across soil, water, and the food web.^3,10,11^

Ingestion is one of the principal routes of exposure, with evidence indicating that MNPs can be efficiently absorbed within the gastrointestinal tract (GIT).^12–15^ Human biomonitoring studies have revealed the presence of MNPs in critical organs, including the liver, kidneys, heart, spleen, brain, human breast milk, and infant feces, as well as reproductive tissues such as the placenta, uterus, testes, and ovaries,^14,16–21^ confirming the ability of MNPs to bypass biological barriers and become systemic. Toxicological assessments in *in vitro* small intestinal epithelium (SIE) models and animal studies have demonstrated that MNPs are taken up by the SIE and can induce cytotoxic effects, including reduced cellular viability, oxidative stress, inflammation, morphological changes, lysosomal dysfunction, DNA damage, induction of apoptotic pathways, metabolic disturbances, and impairment of cellular barrier function.^22–29^

Recent advances in the physicochemical assessment of MNPs highlight a significant discrepancy between the properties of commercially available primary MNPs, predominantly polystyrene (PS), and occasionally polyethylene (PE) and poly­(vinyl chloride) (PVC), and those encountered in natural environments.^1,22–26,30^ These laboratory-grade particles are typically characterized as monosized, symmetrical, or spherical, possessing surface chemistries reflective of their respective polymers. Unlike their primary counterparts, secondary MNPs, generated by environmental forces and municipal waste processing, exhibit irregular morphologies, diverse size distributions, distinct surface chemistries, and heterogeneous surface topologies. They often possess altered surface chemistries resulting from UV photo-oxidation, which include bioreactive oxygen-containing functional groups.^1,31^ Given that the physicochemical properties of MNPs fundamentally influence their biological interactions, the toxicological profiles of environmentally relevant MNPs are expected to differ considerably from those of primary MNPs.^32,33^ This oversight has perpetuated significant knowledge gaps regarding the lifecycle impacts of widely used plastics and the associated generated MNPs, which take place during postweathering/aging and incineration.^34,35^

Recent investigations have also highlighted the ability of MNPs to sorb and concentrate environmental pollutants (EPs), such as toxic elements and organic contaminants.^36–38^ This phenomenon raises significant concerns regarding the agricultural implications of plastic contamination in soil, water, and the food chain, given that plants were reported to take up and bioaccumulate MNPs from soil or water.^39–41^ We recently found that 25 nm PS NPs increase the bioavailability of arsenic in a small intestinal epithelium (SIE) model, as well as in edible shoots of lettuce.^39,42^ Despite these efforts, critical questions remain unanswered, including the capacity of environmentally relevant aged and incinerated MNPs to sorb harmful EPs, form EP corona on MNP surface, and whether coexposure to these substances impacts gastrointestinal toxicity to a greater extent and affects the bioavailability of EPs and MNPs.

To address these knowledge gaps, we have recently synthesized and extensively characterized environmentally relevant PVC MNPs to facilitate a comprehensive risk assessment analysis throughout their lifecycle, which includes processes such as mechanical fragmentation/cryomilling, photo-oxidation/aging, and incineration.^43^ PVC in the waste phase is highly susceptible to degradation, resulting in PVC MNPs accounting for approximately 10.3% of MNP pollution in terrestrial ecosystems, ranking third after polypropylene and PE.^44–46^

In this study, we investigated the biointeractions, toxicity, and bioavailability of PVC MNPs across the plastic waste lifecycle, both alone and under coexposures with EPs, using an *in vitro* transwell triculture SIE model, combined with an *in vitro* three-phase (oral, gastric, and small intestinal) simulated digestion to replicate biotransformations throughout the GIT. The sorption of EPs by PVC MNPs in water and across the GIT and the translocation of EPs (alone or sorbed to PVC MNPs) through the SIE were quantified using inductively coupled plasma mass spectrometry (ICP-MS) and liquid chromatography–mass spectrometry (LC-MS). Translocation of PVC MNPs through the SIE was quantified by pyrolysis–gas chromatography–mass spectrometry (Py-GCMS). Lastly, RNA sequencing was performed to elucidate the gene expression. The study design is illustrated in Figure 1.

**Figure 1:**
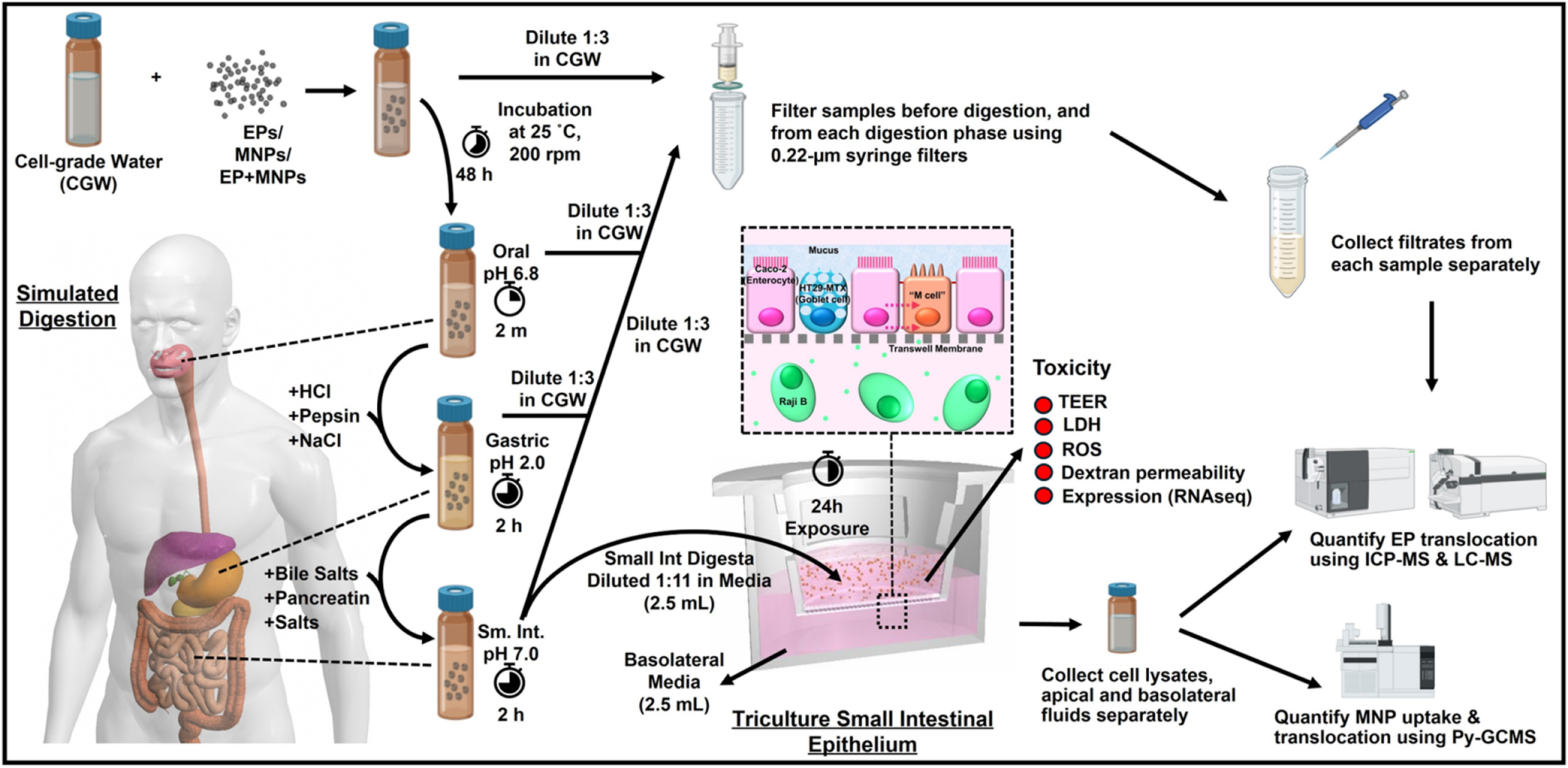
Study design overview.

## 2 Materials and Methods

### 2.1 PVC MNPs

The PVC MNPs were generated to mimic the three primary lifecycle degradation scenarios: mechanical degradation/cryomilling, followed by UV photo-oxidation, and incineration of plastic at the end of life. Test MNPs included cryomilled and unaged PM_10_ (PVC-UA), cryomilled and UV-aged PM_10_ (PVC-A), and PM_0.1_ incinerated (PVC-I) MNPs. Details on the synthesis and physicochemical characterization of these PVC MNPs are presented in our companion publication by Das et al.^43^

### 2.2 Preparation of PVC MNPs and PVC MNP + EP Suspensions in a Food Model (Water)

EPs: As­(V)_2_O_5_ (product number: 255459), Pb­(II)­(NO_3_)_2_ (product number: 228621), Cr­(III)­Cl_3_·6­(H_2_O) (product number: 230723), boscalid (product number: 33875), and PFOS (product number: 93497) used in this study were purchased from Sigma-Aldrich, MO, USA. Cell-grade water (CGW) (product number: SH30529.03) was purchased from HyPure, Cytiva, USA. The starting concentrations of MNPs (PVC-UA, PVC-A, and PVC-I), EPs (As, Pb, Cr, boscalid, and PFOS), and MNPs mixed with EPs (EP-PVC-UA, EP-PVC-A, and EP-PVC-I) suspended in a fasting food model (water in this case) are provided in Supporting Table 1. The suspensions of PVC MNPs alone and PVC MNP + EP mixtures in water were vortexed for 30 s and then water-bath sonicated for 5 min to ensure a stable and homogeneous dispersion.^43,47^ The justification for the selection of target oral concentrations (TOCs) of PVC MNPs and EPs is provided in Supporting Information 1.

### 2.3 Assessment of EP Sorption by PVC MNPs in Water

The sorption of EPs by PVC MNPs was assessed by measuring free EPs in filtrates of MNP+EP suspensions. Starting MNP+EP suspensions were incubated for 48 h at room temperature (RT) on an orbital shaker (VWR, PA, USA) at 200 rpm to allow the partitioning and equilibration of EPs with MNPs. The suspensions were then filtered using regenerated cellulose filter tubes (Thermo Fisher Scientific, MA, USA). The concentration of As, Pb, and Cr after filtration was quantified by ICP-MS using an Agilent 7850x ICP-MS (Agilent Technologies, Inc., Santa Clara, CA, USA) (Supporting Information 2). The concentration of boscalid and PFOS after filtration was quantified using a 1290 ultraperformance liquid chromatograph (Agilent) coupled with a SciEx 7500 triple-quadrupole mass spectrometer (LC-MS) (Supporting Information 2). The percentage of EPs sorbed was calculated using eq 1.^42^

1 $$\%\;\mathrm{EP}\;\mathrm{sorption}\;\mathrm{by}\;\mathrm{MNPs}=100\times \frac{\mathrm{total}\;\mathrm{EP}-\mathrm{free}\;\mathrm{EP}}{\mathrm{total}\;\mathrm{EP}}$$

where “Total EP” is the starting concentration of EPs initially mixed with MNPs in water, and “Free EP” is the concentration of EPs in the filtrate.

### 2.4 *In Vitro* Simulated Digestion of MNPs, EPs, and MNP + EP Mixtures

A three-phase (oral, gastric, and small intestinal) *in vitro* simulated digestion was conducted as detailed previously.^29,42,48,49^ A detailed method is provided in Supporting Information 3.

### 2.5 Assessment of the Fate of EPs Sorbed on PVC MNPs across the GIT

The sorption of EPs by MNPs in the oral, gastric, and small intestinal (SI) phases of simulated digestion was quantified via ICP-MS and LC-MS analysis (Supporting Information 2). The percentage of EPs sorbed on PVC MNPs across the GIT was calculated using eq 1:

The percentage loss of EPs across the simulated digestion stages, including losses during pipetting, laboratory consumable use, and through filter tubes, was determined. More details are provided in Supporting Information 2 and Supporting Information Figure 1.

### 2.6 Colloidal Characterization of SI Digesta of Water Containing PVC MNPs and PVC MNP + EP Mixtures

A multiangle laser diffraction (MALD) particle size analyzer (Mastersizer 3000, Malvern Instruments, Ltd.) equipped with a wet dispersion unit (Hydro SV, Malvern Panalytical, Sunnyvale, CA, USA) was used for these analyses. The 633 and 466 nm laser sources were used to measure the volume-weighted particle size distributions of SI digesta of water containing 600 μg/mL TOC of PVC MNPs and PVC MNPs coexposed with EPs, while stirring at 1800 rpm.^50^ The volume-weighted size distributions were averaged using the built-in software (Mastersizer Xplorer v5.30).

### 2.7 Preparation of *In Vitro* Triculture SIE Model and Exposure to MNP, EP, and MNP + EP Digestas for Toxicological Analysis

The preparation of the *in vitro* triculture SIE model and exposure studies were conducted as detailed previously.^29,42,48,49^ Details of the methods are provided in Supporting Information 3.

The reactive oxygen species (ROS) production, lactate dehydrogenase (LDH) release, trans-epithelial electrical resistance (TEER), and dextran permeability were assessed as previously described.^42^ Details of the methods are provided in Supporting Information 4.

### 2.8 Quantification of EP Translocation across the SIE

The method for sample collection after a 24 h exposure period is provided in Supporting Information 2. The concentrations of each toxic element and organic pollutant in the initial digesta-media mixtures and final apical and basolateral fluids were assessed using ICP-MS and LC-MS, respectively (Supporting Information 2). The % EP translocation was determined using the following eq 2

2 $$\%\;EP\;translocation=\left(100\times \frac{final\;BL\;EP\;concentration\times BL\;vol.}{starting\;apical\;EP\;conentration\times apical\;vol.}\right)$$

where “Final BL EP concentration” refers to the concentration of EPs measured from the basolateral compartment of the transwell plate, after 24 h of exposure; “BL vol.” refers to the volume of media added to the basolateral compartment; “Starting apical EP concentration” refers to the initial concentration of EPs mixed with MNPs measured from the apical compartment of the transwell plate; and “Apical vol.” refers to the total volume of digesta added to the apical compartment of the transwell plate.

The percentage loss of EPs in the transwell SIE model over a 24 h period for the translocation assessment was determined. More details are provided in Supporting Information 2 and Supporting Figure 1.

### 2.9 Quantification of PVC MNP Uptake and Translocation in the SIE

The details on the sample collection and quantification of the uptake and translocation of PVC MNPs across the SIE using Py-GCMS are provided in Supporting Information 5. The percentage of MNP uptake and translocation was calculated using the following eqs 3 and 4.

3 $$\%\;\mathrm{MNP}\;\mathrm{uptake}=100\times \frac{\mathrm{lysate}\;\mathrm{MNP}\;\mathrm{concentration}\times \mathrm{lysate}\;\mathrm{vol}.}{\mathrm{starting}\;\mathrm{apical}\;\mathrm{MNP}\;\mathrm{conentration}\times \mathrm{apical}\;\mathrm{vol}.}$$

4 $$\%\;\mathrm{MNP}\;\mathrm{translocation}=100\times \frac{\mathrm{final}\;\mathrm{BL}\;\mathrm{MNP}\;\mathrm{concentration}\times \mathrm{BL}\;\mathrm{vol}.}{\mathrm{starting}\;\mathrm{apical}\;\mathrm{MNP}\;\mathrm{conentration}\times \mathrm{apical}\;\mathrm{vol}.}$$

where “Lysate MNP concentration” refers to the levels of PVC MNPs in cell lysates; “Lysate vol.” refers to the total volume of cell lysates collected for analysis; “Final BL MNP concentration” refers to the levels of PVC MNPs measured from the basolateral compartment of the transwell plate, after 24 h of exposure; “BL vol.” refers to the volume of media added to the basolateral compartment; “Starting apical MNP concentration” refers to the initial levels of PVC MNPs quantified from the apical compartment of transwell plate; and “Apical vol.” refers to the refers to the total volume of digesta added to the apical compartment of the transwell plate.

### 2.10 Assessment of Effects on Gene Expression (RNA-seq)

The samples for RNA sequencing (RNA-seq) expression analysis were collected as previously detailed.^51^ More details are provided in Supporting Information 6.

### 2.11 Statistical Analysis

Toxicological experiments were performed with a sample size (N) of 6 for each treatment. EP-sorption studies, uptake, and translocation experiments were performed with a sample size of 3 for each treatment. Gene expression analysis was performed in triplicate. Statistical analysis was performed in GraphPad Prism 10.4.2 software (GraphPad Software, Inc., San Diego, CA). Results of toxicological, uptake, and translocation experiments were analyzed by one-way ANOVA with Dunnett’s multiple comparisons test.

## 3 Results and Discussion

### 3.1 Physicochemical Properties of PVC MNPs across Their Lifecycle

The cryomilled (PVC-UA, termed unaged), cryomilled and UV-photooxidized (PVC-A, termed aged), and incinerated (PVC-I) PVC MNPs used in this study were previously synthesized and characterized in detail by Das et al.^43^ A summary of the properties of PVC MNPs is provided in Supporting Information 7, Supporting Figures 2A,B, 3, 4, and 5, and Supporting Tables 3 and 4.

### 3.2 Sorption of EPs by PVC MNPs in Water

The results of EP-sorption by PVC MNPs in water are summarized in Figure 2A and Supporting Information Table 5. In general, all three lifecycle-specific PVC types sorbed substantial amounts of both toxic elements and organic pollutants to varying extents. PVC-A sorbed most toxic elements to a significantly greater extent than PVC-UA and PVC-I MNPs. Specifically, PVC-A sorbed 21.7% of Cr, which did not differ significantly from Cr sorption by PVC-UA (18.6%) but was 126.8% higher (*p* < 0.001) than Cr sorption by PVC-I MNPs (9.5%). PVC-A sorbed 27.2% of As, which was 35.7% (*p* < 0.01) and 80.4% (*p* < 0.001) higher than As sorption by PVC-UA (20.0%) and PVC-I (15.0%), respectively. The sorption of Pb by PVC-A (29.9%) was 31.4% (*p* < 0.01) and 23.8% (*p* < 0.01) higher than the sorption by PVC-UA (22.8%) and PVC-I (24.1%), respectively.

**Figure 2:**
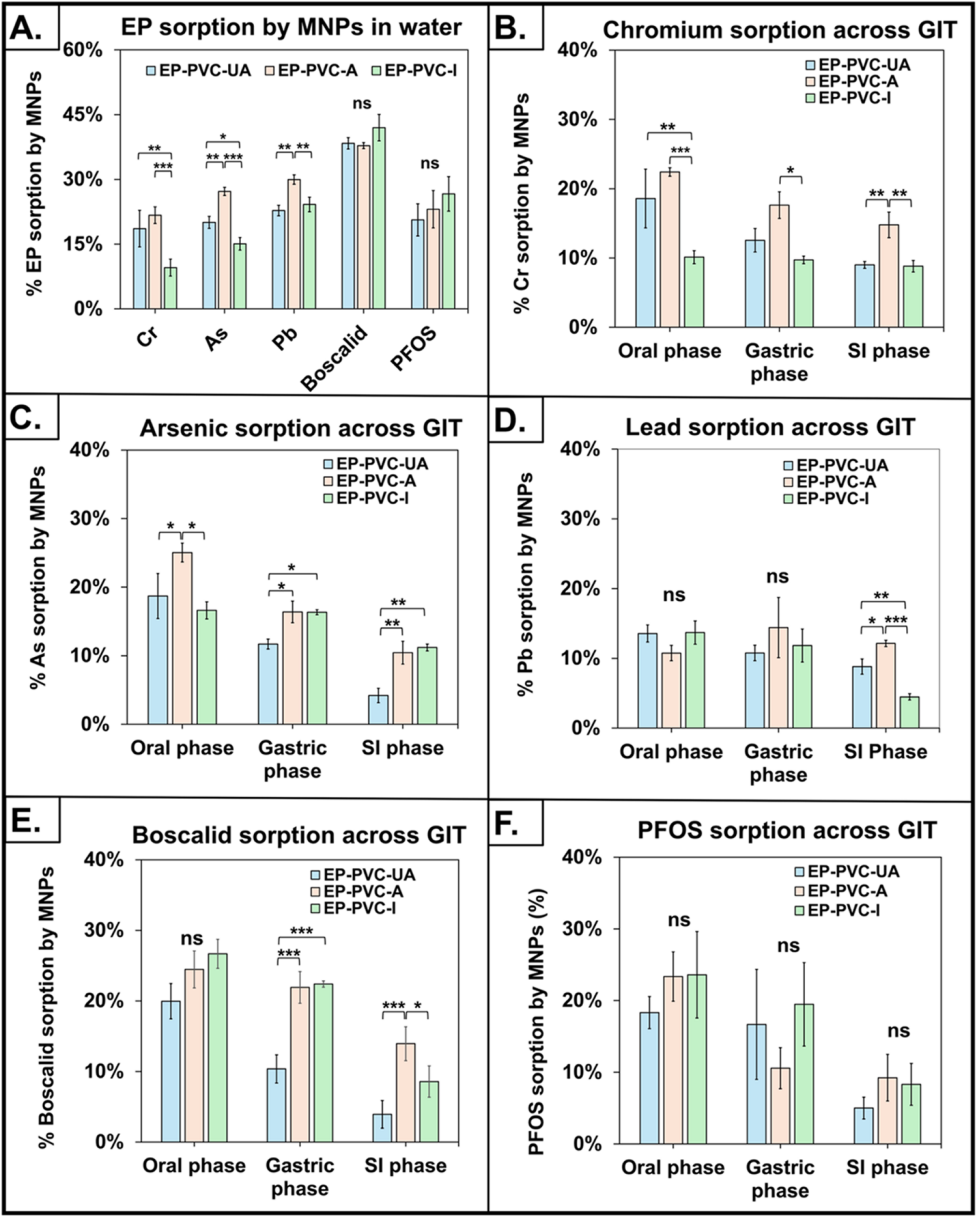
Sorption of EPs by PVC MNPs in water and across the GIT. (A). Sorption of EPs by PVC MNPs in water. (B). Sorption of Cr by PVC MNPs across the GIT. (C). Sorption of As by PVC MNPs across the GIT. (D). Sorption of Pb by PVC MNPs across the GIT. (E). Sorption of boscalid by PVC MNPs across the GIT. (F). Sorption of PFOS by PVC MNPs across the GIT. Data are shown as mean ± SD, ns = nonsignificant, *p < 0.05, **p < 0.01, and ***p < 0.001.

Together, these findings indicate that the sorption of toxic elements by PVC MNPs is MNP lifecycle-stage- and element-specific and that aged PVC MNPs generally sorb toxic elements to a greater extent than their fragmented and unaged (PVC-UA) and incinerated (PVC-I) counterparts.

No significant differences in the sorption of organic pollutants (boscalid and PFOS) were observed among the different PVC MNPs. PVC-UA, PVC-A, and PVC-I sorbed 37.8%, 38.3%, and 41.9% of boscalid and 23.0%, 20.6%, and 26.6% of PFOS, respectively. Thus, sorption of organic pollutants did not appear to be MNP lifecycle stage-specific. However, sorption of organic EPs was EP-specific, with boscalid being sorbed to a greater extent than that of PFOS.

### 3.3 Fate of EPs Sorbed on PVC MNPs across the GIT

The fate of EPs sorbed on PVC MNPs across the GIT is summarized in Figure 2B–F and Supporting Information Table 6. In general, EP sorption levels across oral, gastric, and small intestinal (SI) phases were both EP- and MNP lifecycle stage-specific, with most EP levels on MNPs decreasing at the SI phase compared to the gastric phase, pointing to desorption/resorption, which can be explained by different pH levels and enzymes across the GIT compartments.

Sorption of Cr by PVC-UA and PVC-A decreased across the three phases of digestion, while sorption of Cr by PVC-I remained nearly constant (Figure 2B). In the oral phase, sorption of Cr by PVC-UA (18.5%) and PVC-A (22.4%) was significantly greater than that of PVC-I (10.1%), by 83.6% (*p* < 0.01) and 121.5% (*p* < 0.001), respectively, with no significant difference between sorption of Cr by PVC-UA and PVC-A. In the gastric phase, sorption of Cr by PVC-A (17.6%) was significantly greater than that of PVC-I (9.7%), by 81.2% (*p* < 0.05), but there was no significant difference between sorption of Cr by PVC-UA and PVC-A. In the SI phase, sorption of Cr by PVC-A MNPs was significantly greater than sorption by PVC-UA (9%) and PVC-I (8.8%) by 64.1% (*p* < 0.01) and 67.7% (*p* < 0.01), respectively.

From the oral to the gastric phase of simulated digestion, the percentage of Cr sorbed by PVC-A MNPs decreased by 27.1% (*p* < 0.05), while no significant change in Cr sorption by either PVC-UA or PVC-I was observed. From the gastric to the SI phase, sorption of Cr by PVC-UA decreased by 39.4% (*p* < 0.05), while no significant changes in sorption of Cr by either PVC-A or PVC-I were noted. Collectively, these results indicate that Cr was desorbed by PVC-A primarily during the gastric phase and by PVC-UA during the SI phase, indicating a corresponding increase in Cr bioaccessibility in the GIT.

Sorption of As by all PVC MNPs was roughly the same in the oral phase of digestion as in water but subsequently decreased across the gastric and SI phases of digestion (Figure 2C). In the oral phase, the sorption of As by PVC-A (25.0%) was significantly greater than that of PVC-UA (18.7%) and PVC-I (16.6%) by 33.8% (*p* < 0.05) and 50.7% (*p* < 0.05), respectively, although there was no significant difference between the sorption of As by PVC-UA and PVC-I. In the gastric phase, sorption of As by PVC-A (16.3%) and PVC-I (16.3%) was significantly greater than that of PVC-UA by 40% (*p* < 0.05) and 39.7% (*p* < 0.05), respectively. Likewise, in the SI phase, sorption of As by PVC-A (10.4%) and PVC-I (11.2%) was significantly greater (*p* < 0.01) than sorption of As by PVC-UA (4.1%) by 148.9% and 167%, respectively.

From the oral to the gastric phase, the percentage of As sorbed by PVC-A and PVC-UA MNPs decreased by 52.8% (*p* < 0.01) and 59.9% (*p* < 0.05), respectively, while no significant change in As sorption by PVC-I was observed. From the gastric to the SI phase, sorption of As by PVC-A, PVC-UA, and PVC-I decreased by 56.9% (*p* < 0.05), 179% (*p* < 0.001), and 46% (*p* < 0.05), respectively. The desorption of As from all PVC MNP types during digestion indicates a corresponding increase in the As bioaccessibility.

Sorption of Pb by PVC MNPs was generally lower in all GIT digestion phases than in water and was, for the most part, unchanged across the GIT (Figure 2D). In the oral phase, there were no significant differences among the sorption of Pb by PVC-UA (13.6%), PVC-A (10.8%), and PVC-I (13.7%). Likewise, in the gastric phase, there were no significant differences among the sorption of Pb by PVC-UA (10.8%), PVC-A (14.4%), and PVC-I (11.8%) MNPs. However, in the SI phase, sorption of Pb by PVC-A (12.1%) was significantly higher than sorption by PVC-UA (8.8%) and PVC-I (4.4%) by 37.6% (*p* < 0.05) and 171.6% (*p* < 0.001), respectively.

From the oral to the gastric phase, the percentages of Pb sorbed by PVC-UA, PVC-A, and PVC-I showed no significant changes. Likewise, there were no significant changes in the sorption of Pb by PVC-UA and PVC-A between the gastric and SI phases. However, sorption of Pb by PVC-I decreased by 164.8% (*p* < 0.001) between the gastric and SI phases. These findings indicate that most of the Pb initially sorbed in water by PVC-I becomes free and thus bioaccessible by the time it reaches the small intestine.

Sorption of boscalid by PVC MNPs generally decreased between water and the oral phase of digestion and decreased further at each subsequent phase across the GIT (Figure 2E). In the oral phase of digestion, as in water, there were no significant differences between the sorption of boscalid by PVC-UA (20.0%), PVC-A (24.4%), and PVC-I (26.6%). In the gastric phase, sorption of boscalid by PVC-A (21.9%) and PVC-I (22.3%) was significantly greater than that of PVC-UA (10.4%) by 111.4% (*p* < 0.001) and 116% (*p* < 0.001), respectively. In the SI phase, sorption of boscalid by PVC-A (13.9%) was higher than that by PVC-UA (3.9%) and PVC-I (8.5%) by 255.5% (*p* < 0.001) and 62.6% (*p* < 0.05), respectively.

Between the oral and gastric phases, sorption of boscalid by PVC-UA decreased by 92.5% (*p* < 0.01), while sorption by PVC-A and PVC-I decreased slightly but not significantly. Between the gastric and SI phases, sorption of boscalid by PVC-UA, PVC-A, and PVC-I MNPs decreased by 164.3% (*p* < 0.01), 57.1% (*p* < 0.05), and 161.2% (*p* < 0.01), respectively. A decreased level of sorption of boscalid during digestion indicates a corresponding increase in the bioaccessibility of boscalid.

Sorption of PFOS by PVC MNPs remained at about the same levels observed in water during the oral phase of digestion but decreased sharply in subsequent phases of digestion (Figure 2F). In the oral phase, PVC-UA (18.3%), PVC-A (23.3%), and PVC-I (23.6%) MNPs showed no significant difference in PFOS sorption. Similarly, in the gastric phase, no difference in PFOS sorption was noted among PVC-UA (16.6%), PVC-A (10.5%), and PVC-I (19.4%) MNPs. In the SI phase, PVC-UA (5%), PVC-A (9.2%), and PVC-I (8.3%) showed no change in the level of PFOS sorption.

Between the oral and gastric phases, sorption of PFOS by PVC-A decreased by 121% (*p* < 0.01), while sorption by PVC-UA and PVC-I did not significantly change. In contrast, between the gastric and SI phases, PFOS sorption by PVC-A did not change significantly, while sorption by PVC-UA and PVC-I decreased by 233.5% (*p* < 0.001) and 134.2% (*p* < 0.01), respectively. Together, these results indicate that sorbed PFOS is desorbed by PVC-A primarily during the gastric phase and by PVC-UA and PVC-I during the SI phase, indicating a corresponding increase in PFOS bioaccessibility.

One potential mechanism by which MNPs may influence the uptake of EPs by SIE is the “Trojan horse” mechanism,^42,52^ whereby EPs are transported across the SIE on the surface of MNPs.^53,54^ Our results confirm that the sorption dynamics of EPs by PVC MNPs in water and across the GIT are dependent on pollutant type and the lifecycle stage of PVC MNPs. Notably, the EP sorption by the MNPs types, especially in the SI phase, was lower than that in water and oral and gastric phases, which suggests that EPs sorbed by the PVC MNPs may be released in the small intestine, thereby increasing their bioaccessibility and ultimately their absorption (bioavailability) in the intestine independent of MNPs. Despite considerably lower sorption of EPs by MNPs in the SI phase, the fraction of EPs that remained sorbed could be cotransported across the SIE.

More alarmingly, aged PVC MNPs generally sorbed higher amounts of EPs than unaged and incinerated MNPs, both in water and in the final SI phase of digestion. This is most likely due to the significant differences in surface chemistry, particularly the increase in oxygen-containing groups, that occur during UV-aging. The toxic elements Cr, and Pb are positively charged and can form complexes with negatively charged functional groups, such as carboxyl and carbonyl groups.^55^ Notably, we reported earlier that UV-aging resulted in a further decrease in the ζ-potential of PVC-A to −33 mV.^43^ This alteration, along with photo-oxidation upon UV-aging, has been reported to enhance the adsorption capacity of MNPs for toxic elements through mechanisms of electrostatic attraction and ion complexation.^55^ In contrast, the influence of photo-oxidation on the adsorption of organic pollutants is more complex and exhibits variability, as observed in our study, and is governed primarily by hydrophobic partitioning and electrostatic interactions. Specifically, PVC-A MNPs sorbed a higher amount of boscalid in water and the SI phase, but no significant changes were observed in the sorption of PFOS. Boscalid is a neutral, moderately hydrophobic compound (log *K*_oW_ ≈ 2.9), and its enhanced sorption to aged PVC MNPs is therefore most plausibly driven by increased surface roughness and oxygen-containing functional groups that facilitate hydrogen bonding and noncovalent interactions, in addition to hydrophobic partitioning. Contrastingly, PFOS is an anionic pollutant with high aqueous stability and strong hydration of its sulfonate headgroup, which likely limits its sensitivity to changes in surface oxidation and ζ-potential of PVC MNPs. Additional interactions, such as π–π stacking, may also contribute; however, their influence is expected to be secondary due to the limited aromatic surface density of PVC.^55^ Overall, our findings indicate that UV-aging PVC MNPs selectively enhance the sorption of organic pollutants, depending on pollutant charge, hydrophobicity, and molecular structure. Further studies are required to confirm the mechanisms underlying the interactions between PVC MNPs and EPs across the GIT.

### 3.4 Particle Size Distribution of PVC MNPs and PVC MNP + EP Mixtures in Water and SI Digesta

The results of the size distribution of PVC MNPs alone and coexposed to EPs in water and SI digesta are presented in Supporting Figure 6A,B. PVC-UA and PVC-A MNPs, alone and coexposed to EPs suspended in water, had a relatively uniform particle size distribution, with D_90_ values ranging between 7.1 and 9.0 μm (Supporting Table 7). Similarly, PVC-I alone and coexposed to EPs also exhibited a uniform size distribution with sharp peaks and D90 values of 0.5 and 1.2 μm, respectively.

In the SI phase of simulated digestion, PVC-UA and PVC-A MNPs alone and coexposed to EPs exhibited broad peaks with a D_90_ value ranging between 16.9 and 19 μm, while PVC-I and EP-PVC-I had relatively sharper peaks with a D_90_ value of 7.0 and 8.4 μm, respectively. The shift in particle size distribution in PVC MNPs and PVC MNP + EP mixtures in comparison to their suspension in water suggests interaction of digestive proteins and enzymes in the SI phase of simulated digestion (*e.g*., mucin, pepsin, pancreatic enzymes), which changes the size distribution of the particles in the digesta.^50^ Such interactions and size shifts may affect the biological fate and toxicological properties of coingested PVC MNPs and EPs.

### 3.5 *In Vitro* Toxicity of PVC MNPs in the Triculture SIE Model

The results of the toxicological assessment of EPs alone, PVC MNPs alone, and the mixture of MNP + EPs in the transwell triculture SIE model are presented in Figure 3. Exposure of the transwell SIE to digestas of EPs alone, or of PVC MNPs across the lifecycle stages, at TOCs of either 200 (corresponding to 16.5 μg/mL of MNPs applied to SIE) or 600 μg/mL (corresponding to 50 μg/mL of MNPs applied to SIE), with or without EPs, caused no significant cytotoxicity (Figure 3A). Likewise, none of the EP or PVC MNP ± EP exposures had a significant effect on TEER, an indicator of epithelial barrier integrity (Figure 3B), or on permeability to either 3 kDa or 70 kDa dextran, indicators of transcellular and paracellular permeability, respectively (Figure 3C,3D). These findings suggest that, at the concentrations tested, PVC MNPs across their lifecycle, exposed alone or with the EPs tested, are minimally toxic and have no significant effect on barrier function in the SIE. The low toxicity observed in the SIE model was expected, given the equivalent low concentrations applied to the SIE cells (50 and 16.5 μg/mL).

**Figure 3:**
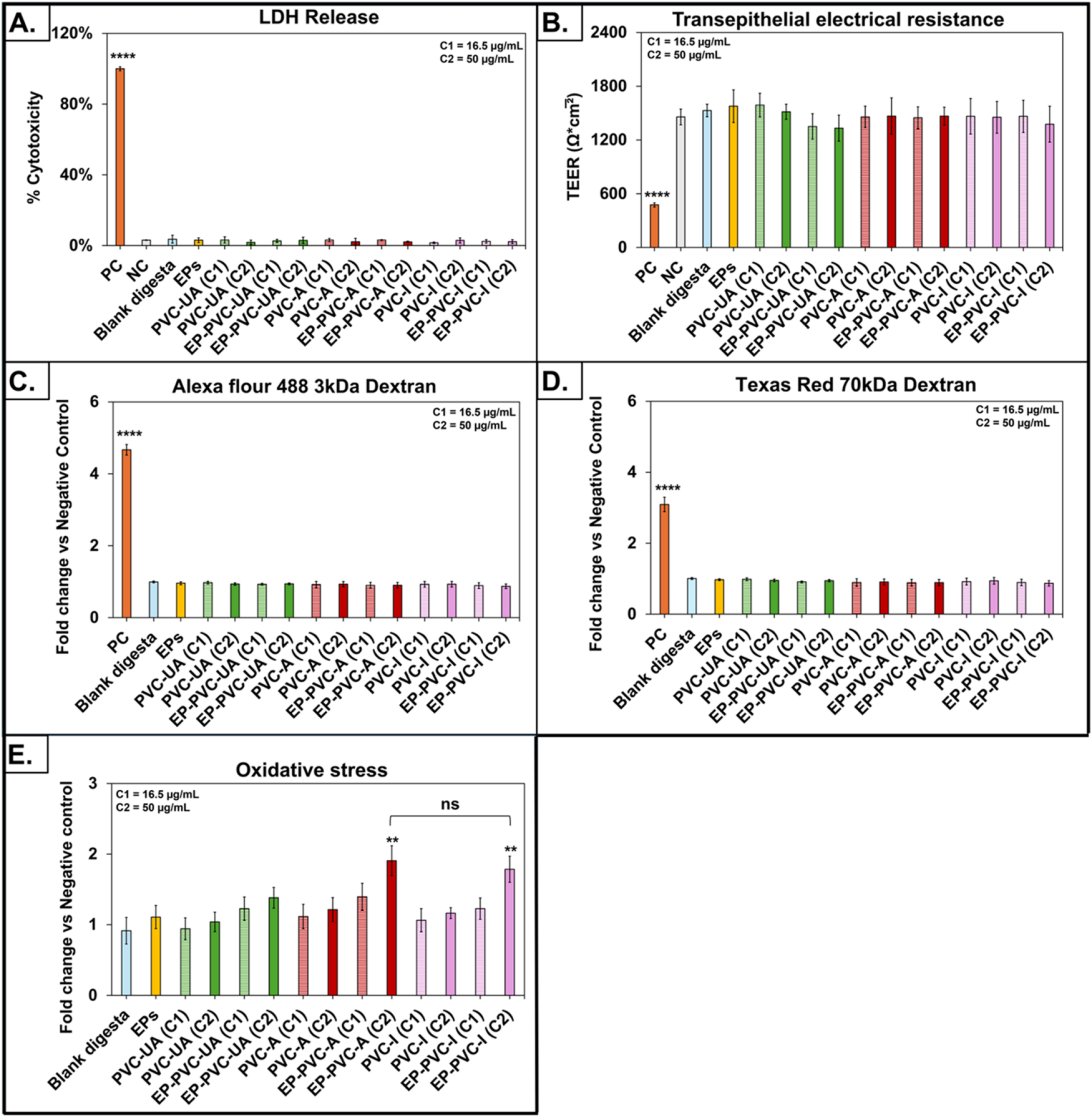
Toxicity assessment of PVC MNPs with and without EPs in the SIE model. (A). Percent cytotoxicity (percent of LDH release relative to that of lysed control cells) after 24 h exposure. (B). TEER after 24 h exposure. (C). Fold change in apparent permeability coefficient (*P*_app_) assessed with fluorescent-labeled Alexa Fluor 488 3 kDa dextran. (D). Fold change in *P*_app_ assessed with fluorescent-labeled Texas Red 70 kDa dextran. (E). Fold change in oxidative stress after 6 h exposure. Each test groups was compared with the blank digesta. Data are shown as mean ± SD, ns = nonsignificant, **p < 0.01, and ****p < 0.0001. Concentration of MNPs applied to SIE: C1 = 16.5 μg/mL (corresponding to 200 μg/mL TOC) and C2 = 50 μg/mL (corresponding to 600 μg/mL TOC).

Exposure to SI digesta of PVC-A and PVC-I MNPs at 600 μg/mL TOC and in the presence of EPs increased ROS production by 108.3% (*p* < 0.01) and 95.1% (*p* < 0.01), respectively, compared to cells exposed to blank digesta. (Figure 3E). These findings also suggest that ROS production is dependent on the lifecycle stage of MNPs, their concentration, and the presence of EPs. The oxidative stress caused by exposure to PVC-A and PVC-I MNPs in the presence of EPs could also be attributed to synergistic interactions between the EPs and these PVC MNPs. Further mechanistic studies are needed to delineate the underlying processes.

### 3.6 Effect of PVC MNPs on Translocation of EPs across the SIE

The effects of the presence of PVC MNPs on the translocation of EPs across the SIE from potential coexposures are summarized in Figure 4 and Supporting Information Table 8. In general, the effects of PVC MNPs on EP translocation were dependent upon the lifecycle stage and concentration of PVC MNPs and the EP type.

**Figure 4:**
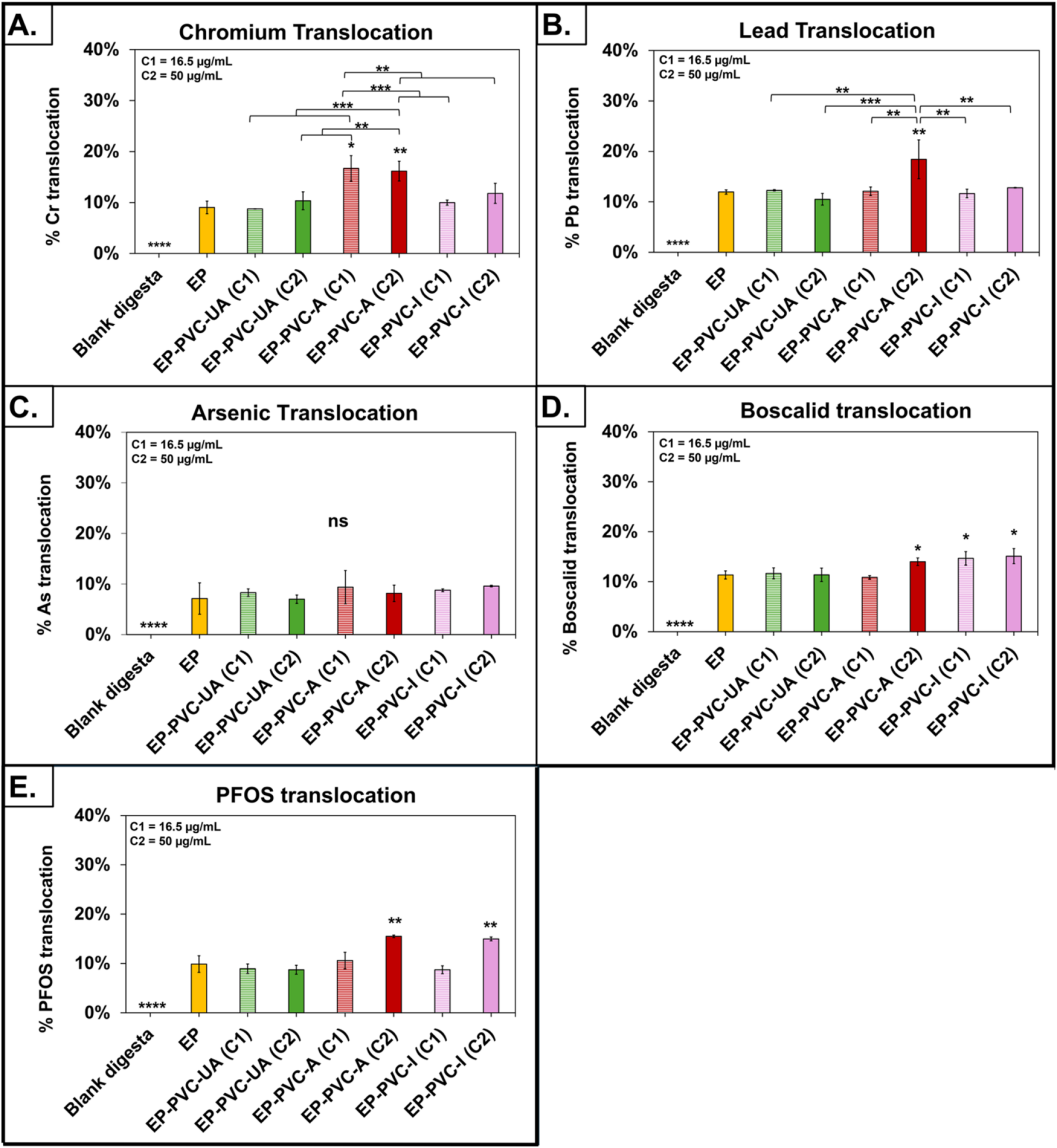
Effect of PVC MNPs on the translocation of EPs across SIE. (A). Effects of PVC MNPs on Cr translocation. (B). Effects of PVC MNPs on As translocation. (C). Effects of PVC MNPs on Pb translocation. (D). Effects of PVC MNPs on boscalid translocation. (E). Effects of PVC MNPs on PFOS translocation. Each test groups was compared with the EPs alone. Data are shown as mean ± SD, *p < 0.05, **p < 0.01, ***p < 0.001, and ****p < 0.0001. Concentration of MNPs applied to SIE: C1 = 16.5 μg/mL (corresponding to 200 μg/mL TOC) and C2 = 50 μg/mL (corresponding to 600 μg/mL TOC).

Notably, the lifecycle “age” of PVC MNP plays a significant role. Specifically, the presence of PVC-A MNPs dramatically increased translocation of Cr by 84.7% (*p* < 0.05) at 200 μg/mL TOC (corresponding to 16.5 μg/mL of MNPs applied to SIE) and by 78.8% (*p* < 0.01) at 600 μg/mL TOC (corresponding to 50 μg/mL of MNPs applied to SIE) compared to EPs alone, whereas PVC-UA and PVC-I had no effect on Cr translocation (Figure 4A). Specifically, translocation of Cr coingested with PVC-A at 200 and 600 μg/mL TOCs through the triculture SIE was 16–17%, while that of Cr alone was 9%. This suggests that effects of PVC MNPs on Cr translocation are lifecycle stage-specific, and that UV-aging is the primary determinant of this effect.

Similarly, the presence of PVC-A at 600 μg/mL TOC increased translocation of Pb by 54.6% (*p* < 0.01) from 11.9% to 18.4%, whereas PVC-UA and PVC-I had no effect on Pb translocation across the SIE at either concentration (Figure 4B). These results suggest that the effects of PVC MNPs on Pb translocation are also lifecycle stage-specific, with UV-aging as the primary determinant.

None of the PVC MNPs had a significant effect on the translocation of As, suggesting that the effects of PVC MNPs on toxic element translocation are element- or possibly valence-specific. The translocation for all test samples ranged between 7 and 9.6% (Figure 4C). Previous studies in the SIE models have reported that As uptake occurs by both passive paracellular transport and active transport via the inorganic phosphate transporter NaPiIIb.^56,57^ Our findings suggest that PVC MNPs, across their lifecycle, may not affect the expression or activity of the NaPiIIb transporter at least at the two TOCs examined.

Translocation of boscalid was significantly increased by the presence of either PVC-A or PVC-I, but not PVC-UA (Figure 4D). Specifically, PVC-A at 600 μg/mL TOC increased boscalid translocation by 23.4% (from 11.3 to 14%, *p* < 0.05) and PVC-I at 200 and 600 μg/mL TOCs increased boscalid translocation by 29.2% (from 11.3 to 14.6%, *p* < 0.05) and 33.1% (from 11.3 to 15.1%, *p* < 0.05), respectively, compared to EPs alone. PVC-UA had no significant effect on boscalid translocation at either TOC. There were no significant differences between boscalid translocation levels in the presence of PVC-A at 600 μg/mL, PVC-I at 200 μg/mL, and PVC-I at 600 μg/mL TOC.

Translocation of PFOS, like that of boscalid, was significantly increased in the presence of PVC-A or PVC-I, but not PVC-UA (Figure 4E). PVC-A at 600 μg/mL (but not 200 μg/mL) TOC increased PFOS translocation by 56.9% (from 9.8 to 15.4%, *p* < 0.05), and PVC-I at 600 μg/mL (but not 200 μg/mL) TOC increased PFOS translocation by 51.7% (from 9.8 to 14.9%, *p* < 0.05), compared to EPs alone. PVC-UA had no significant effect on PFOS translocation at either TOC. There was no significant difference between PFOS translocation levels in the presence of PVC-A and PVC-I at 600 μg/mL TOC.

Overall, these results suggested that the translocation of organic pollutants is MNP lifecycle stage-specific and also concentration-dependent. EPs such as Cr, Pb, boscalid, and PFOS have been reported to traverse cell membranes via both passive diffusion and active transport mediated by membrane transporters.^58–62^ However, our findings suggest that although dependent on pollutant type, UV-aged and incinerated PVC MNPs might act as a vehicle in the gastrointestinal milieu to potentiate cellular exposure to EPs. Future studies must delineate the mechanisms underlying the effects of PVC MNPs on the translocation of EPs.

### 3.7 Effect of EPs on the Uptake and Translocation of PVC MNPs

The effects of EPs on the uptake and translocation of PVC MNPs in the SIE model are summarized in Figure 5 and Supporting Table 9. At the lower MNP concentration of 200 μg/mL TOC (corresponding to 16.5 μg/mL of MNPs applied to SIE), uptake of PVC-I was 67.9% greater than that of PVC-UA (*p* < 0.05) and 102.1% greater than that of PVC-A (*p* < 0.05) (Figure 5A). Similarly, in the presence of EPs, uptake of PVC-I was 115.9% greater than that of PVC-UA (*p* < 0.01) and 80.3% greater than that of PVC-A (*p* < 0.001). There were no significant differences between % uptake of PVC-UA and PVC-A, with or without EPs, at either concentration, or between % uptake of any of the PVC MNPs, with or without EPs, at TOC of 200 and 600 μg/mL (corresponding to 50 μg/mL of MNPs applied to SIE). Collectively, these results suggest that uptake of PVC MNPs, with or without EPs, is MNP lifecycle stage-dependent, but not concentration- or EP-dependent.

**Figure 5:**
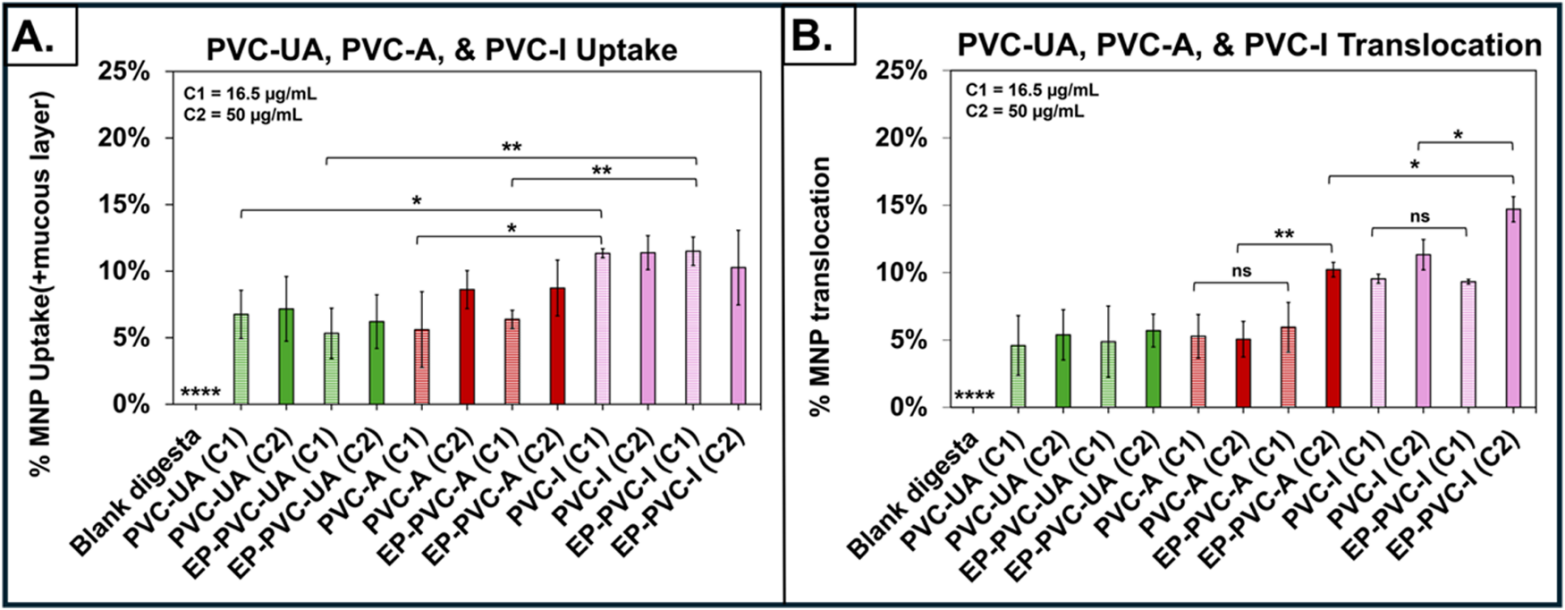
Effects of EPs on the uptake and translocation of PVC MNPs across SIE. (A). Effect of EPs on the uptake of PVC MNPs across SIE. (B). Effect of EPs on the translocation of PVC MNPs by SIE. Data are shown as mean ± SD, *p < 0.05, **p < 0.01, and ****<0.0001. Concentration of MNPs applied to SIE: C1 = 16.5 μg/mL (corresponding to 200 μg/mL TOC) and C2 = 50 μg/mL (corresponding to 600 μg/mL TOC).

The presence of EPs increased the translocation of PVC-A MNPs (at 600 μg/mL TOC) across the SIE by 93.8% (*p* < 0.01) and 102.2% (*p* < 0.01) in comparison to PVC-A MNPs at 200 and 600 μg/mL TOCs without EPs, respectively (Figure 5B). Additionally, translocation of PVC-A (at 600 μg/mL TOC) with EPs was higher than that of PVC-UA (by 122.8, *p* < 0.01 at 200 μg/mL TOC and 89.9%, *p* < 0.05 at 600 μg/mL TOC), of EP-PVC-UA (by 109.9%, *p* < 0.01 at 200 μg/mL TOC and 79.4%, *p* < 0.05 at 600 μg/mL TOC), and of EP-PVC-A (by 71.9, *p* < 0.05 at 200 μg/mL TOC). These results suggest that the translocation of aged PVC MNPs is EP- and concentration-dependent.

No difference (*p* > 0.05) in translocation was observed among PVC-I alone of 200 μg/mL TOCs (9.5%), PVC-I alone of 600 μg/mL TOCs (11.3%), and EP-PVC-I of 200 μg/mL TOCs (9.3%) (Figure 5B). Conversely, the translocation of EP-PVC-I MNPs of 600 μg/mL TOC was higher than that of PVC-I alone (by 54.0%, *p* < 0.05 at 200 μg/mL TOC and 29.7%, *p* < 0.05 at 600 μg/mL TOC) and EP-PVC-I MNPs (by 57.7%, *p* < 0.05 at 200 μg/mL TOC). Collectively, these observations suggest that the translocation of PVC-I at 200 μg/mL TOC is not EP-dependent; however, the translocation of PVC-I at 600 μg/mL TOC is EP-dependent, indicating a concentration-dependent effect.

The observed increase in translocation of aged PVC-A MNPs in the presence of EPs, which was not observed with unaged PVC-UA, is likely due to alterations in the surface chemistry and physicochemical properties of PVC-A caused by UV-aging, including increased surface oxygen-containing functional groups, specific surface area, and surface roughness.^43^ The modified surface properties may facilitate passive diffusion through enhanced membrane permeability or promote specific active uptake mechanisms, such as phagocytosis and clathrin-mediated endocytosis, which are shown in our previous mechanistic studies.^29,51^ Furthermore, the presence of EPs may alter the electrostatic and hydrophobic interactions between the MNPs and cellular membranes as well as the expression of membrane receptors or endocytic pathway genes, potentially enhancing cellular uptake and translocation efficiency.^13,63^

The greater uptake and subsequent translocation observed in PVC-I can be attributed to its unique nanoscale particle size distribution. Unlike unaged and aged PVC MNPs, which are primarily micron-sized particles (less than 10 μm), the majority of PVC-I particles are predominantly smaller than 100 nm, thereby presenting a greater surface area for interaction and absorption with intestinal cells, and nanoscale particles have higher translocation potential compared to micron-sized counterparts.^1,22,64^ Moreover, they contain high levels of PAHs, and the lipid solubility of PAHs is well-documented.^65,66^ It is worth noting that high-molecular-weight PAHs identified in PVC-I are highly hydrophobic and can increase the overall hydrophobicity of the particle surface, potentially enhancing the sorption of other nonpolar organic pollutants via π–π interactions and hydrophobic partitioning. Their propensity for intestinal absorption may significantly enhance the uptake and translocation of particles. However, this hypothesis must be confirmed through mechanistic assessments in the future.

### 3.8 Effects of Aged PVC and Incinerated PVC MNPs and EPs on Triculture SIE Gene Expression

The effects of PVC MNPs and EPs on the SIE gene expression are summarized in Supporting Information Figure 7 and Supporting Information Table 10. In general, both EPs and PVC MNPs reduced (*p* < 0.05) the expression of multiple cell junction genes, which may in part be responsible for the observed reciprocal effects of EPs and PVC MNPs on their translocation across the SIE.

Notably, the expression of several cell junction genes was significantly altered in SIE cells exposed to digestas of PVC-A in the presence of EPs (600 μg/mL TOC) compared to SIE exposed to digestas of EPs alone (Supporting Figure 7A). Specifically, tight junction gene *Cldn5* was downregulated (log_2_ FC = −3.4); adherens junction genes *Notch1* and *Cdh5* were downregulated (log_2_ FC = −1.7) and upregulated (log_2_ FC = +5.3), respectively; gap junction gene *Gja4* was downregulated (log_2_ FC = −2.1); and focal adhesion genes *Itga4* and *Itgal* were downregulated, exhibiting a log_2_ fold changes of −1.60 and −1.14, respectively, in SIE treated with digestas of EPs and PVC-A compared to SIE treated with EPs alone.

Similarly, coexposure of the SIE to digesta of PVC-I (600 μg/mL TOC) with EPs also altered expression of multiple junction genes compared to exposure of EPs alone (Supporting Figure 7B). Specifically, the tight junction gene *Cldn11* was downregulated (log_2_ FC = −4.68); adherens junction genes *Notch1* and *Cdh5* were downregulated (log_2_ FC = −2.4) and upregulated (log_2_ FC = +4.65), respectively; focal adhesion genes *Itgal* and *Itgb7* were downregulated, exhibiting a log_2_ fold changes of −4.38 and −1.75, respectively; and desmosome-related gene *Dsg4* was downregulated (log_2_ FC = −4.91) in SIE treated with digestas of EPs and PVC-I compared to SIE treated with EPs alone.

We identified alterations in gene expression within the classic claudin family, particularly noting a significant downregulation of *cldn5* and *cldn11* upon coexposure to EPs with PVC-A and PVC-I MNPs. These “sealing claudins” are vital for maintaining the charge- and size-selective properties of tight junctions.^67^ Their reduced expression indicates increased paracellular permeability, which may facilitate the translocation of exogenous molecules across the epithelial barrier.^68^ We also observed downregulation of the adherens junction gene *Notch1*, alongside upregulation of the adherens junction gene *Cdh5* upon coexposure to EPs with PVC-A and PVC-I MNPs. Downregulation of *Notch1* is likely to impair cell fate signaling, hinder regenerative capacity, and contribute to barrier fragility.^69,70^ Conversely, the upregulation of *Cdh5* may indicate an adaptive response, potentially reflecting a compensatory mechanism that enhances cell adhesion or a stress-induced reprogramming of epithelial cells. The reduction in *Gja4* expression upon coexposure to EPs with PVC-A MNPs negatively affects the integrity of gap junctional intercellular communication.^71^ A significant reduction in the cell-matrix junction focal adhesion genes, specifically *Itga4*, *Itgal*, and *Itga7* expression, was observed upon coexposure to EPs with PVC-A and PVC-I MNPs. Integrins, which are encoded by the *Itga* genes, serve as the primary epithelial cell surface receptors, linking intracellular actin fibers to the extracellular matrix. PVC-A and PVC-I MNPs could bind to integrins on the apical surfaces of epithelial cells, leading to a temporary and reversible opening of tight junctions, resulting in increased intestinal permeability.^72–74^ Some additional genes, such as the intercellular adhesion gene *Icam2* and the desmosome-related gene *Dsg4*, were specifically downregulated when EPs were coexposed to PVC-I MNPs. *Icam2* is a cell adhesion molecule that maintains intercellular adhesion and junctional organization.^75^ The downregulation of the *Dsg4* gene indicates disruption of desmosomal adhesion, leading to weakened epithelial integrity and compromised barrier function.^76^

Overall, these findings suggest that aged and incinerated PVC MNPs, when coexposed with EPs, could cause weakening of cell junctions and intercellular adhesion, which may result in dysregulation of paracellular transport and, presumably, contribute to the observed increased translocation of EPs in the SIE.

Coexposure of the SIE to the digesta of EPs and PVC-A MNPs (600 μg/mL TOC) also altered (*p* < 0.05) the expression of multiple junction genes compared to exposure of PVC-A (600 μg/mL TOC) (Supporting Figure 7C). Specifically, the tight junction genes *Cldn5*, *Cldn11*, and *Jam3* were downregulated, exhibiting log_2_ fold changes of −5.0, −4.2, and −2.3, respectively. Adherens junction gene *Cdh4* was downregulated (log_2_ FC = −1.3), and gap junction gene *Gja4* was downregulated (log_2_ FC = −6.2).

Similarly, the expression of several cell junction genes was altered (*p* < 0.05) in SIE exposed to digestas of EP-PVC-I (600 μg/mL TOC) compared to exposure to PVC-I (600 μg/mL TOC) (Supporting Figure 7D). Specifically, the tight junction gene *Cldn14* was downregulated (log_2_ FC = −3.8), adherens junction gene *Cdh6* was downregulated (log_2_ FC = −4.5), and desmosome-related gene *Dsg4* was downregulated (log_2_ FC = −6.0).

We observed significant downregulation of tight junction genes, particularly the “sealing claudins” *Cldn5*, *Cldn11*, and *Cldn14*, indicating increased paracellular permeability. We observed a significant downregulation of another tight junction gene, *Jam3*, suggesting disrupted epithelial barrier integrity in cells coexposed to PVC-A MNPs and EPs. Earlier reports have suggested that overexpression of *Jam3* improves tight junctions and restores an epithelial phenotype in lung squamous cell carcinoma cells.^77^ Although *Jam3* is abundantly expressed in intestinal epithelial cells, its precise function remains unclear.^78^ Downregulation of the gap junction gene, *Gja4*, was observed in cells coexposed to PVC-A and EPs. Furthermore, a downregulation of adherens junction genes, *Cdh4* and *Cdh6*, was observed when PVC-A and PVC-I were coingested with EPs, respectively, suggesting a significant disruption of epithelial adhesion, structural integrity, and tissue organization. Several reports have demonstrated the functional roles of these genes in epithelial structure and even in the regulation of epithelial-to-mesenchymal transition-like activity.^79–81^ A significant downregulation of the *Dsg4* gene was observed in cells specifically coexposed to PVC-I MNPs and EPs. Since desmosomes are responsible for cell-to-cell adhesion in epithelial cells, these results suggest a loss of integrity in the intestinal epithelium.^76^

Collectively, these findings suggest that EPs coingested with aged and incinerated PVC MNPs could also dysregulate paracellular transport by negatively impacting cell junctions and intercellular adhesion, contributing to increased PVC MNP translocation in SIE.

### 3.9 Environmental Implications, Limitations, and Future Work

Our study highlights that assessments based solely on pristine micro­(nano)­plastics may underestimate or misrepresent the real-world ecological and health risks of MNPs and that future studies should focus exclusively on environmentally relevant (UV-aged) MNPs. Currently, environmental risk assessments often evaluate MNPs and chemicals separately. This study underscores the need for integrated regulatory frameworks that account for coexposure and lifecycle-derived transformations of MNP materials with other copollutants. Our findings also suggest that managing plastic pollution requires lifecycle-aware approaches that emphasize prevention, reduction, and improved waste handling to minimize the formation of high-affinity aged and thermally altered MNPs in the environment.

Although this study provides valuable insights into MNP research, it is essential to acknowledge several limitations that may affect the comprehensiveness of our findings. First, our investigation did not encompass the binding kinetics between EPs and PVC MNPs. Understanding this interaction is crucial, as it can significantly influence the release rates of EPs during their transition from environmental matrices to biological systems. The absence of a kinetic study across various concentrations limits our ability to elucidate the de/adsorption dynamics of EPs in both environmental and biological contexts over time. A thorough kinetic analysis would enhance our understanding of the temporal behavior of these compounds in the presence of MNPs within the complex microenvironment of the GIT, including potential interactions with digestive enzymes, food matrices, and the formation of hard and soft coronas on the surface of MNPs, including chemical, protein, and lipid coronas. Furthermore, this study focused on EP exposures consisting of a few toxic elements and organic compounds; however, it is important to recognize that real-world environments typically present a much more complex mixture of pollutants occurring simultaneously. This study also did not address the potential leaching of chemicals/additives from the MNPs themselves and their interactions with EPs on environmental or biological matrices. Future investigations should prioritize these factors to better elucidate the environmental and health implications of MNPs

Future studies should also include *in vivo* studies to validate the *in vitro* data and further explore the molecular mechanisms underlying the observed downregulation of junctional proteins, as well as their implications for epithelial barrier function and the resilience of these systems post-exposure. *In vitro* and *in vivo* studies are necessary to evaluate the impact of varying dietary factors on the bioavailability of pollutants in the presence of MNPs. Understanding the interactions between MNPs derived from various polymers and their roles in the bioaccumulation of pollutants will enable accurate risk assessment and aid in developing effective strategies to mitigate their impact on human health and the environment.

## Supplementary Material



## Data Availability

The raw data supporting the conclusions of this article will be made available by the authors upon request.

## Notes

Methods for (1) in vitro triculture SIE model preparation; (2) toxicological analysis, and (3) quantifying toxic elements, organic pollutants, and MNPs. Data on (1) MNP characterization, (2) MNP-EP interaction studies in water and across the GIT, and (3) MNP and EP bioavailability studies (PDF)
