# Supplementary material for: Lifecycle Implications of Poly(vinyl chloride) (PVC) Micro(nano)plastics (MNPs): Interactions with Coexposed Environmental Pollutants (EPs) and Impact on Their Toxicity and Bioavailability
Source: Environ Sci Technol. 2026 May 14;60(20):14248–61. doi: 10.1021/acs.est.5c14500 (PMC13217554; doi:10.1021/acs.est.5c14500)
Supplement: Supplementary file 1 [file es5c14500_si_001.pdf]

Lifecycle Implications of Poly(vinyl chloride) (PVC) Micro(nano)plastics (MNPs): Interactions with Coexposed Environmental Pollutants (EPs) and Impact on Their Toxicity and Bioavailability

Satwik Majumder[1], Glen DeLoid[1], Milton Das[1], Mandeep Kaur[2], Eshun Gaddi[3], Sarah Alotaibi[3], Nubia Zuverza-Mena[2], Omowunmi Sadik[3], Jason White[2], Philip Demokritou[1*]

sm3397@eohsi.rutgers.edu
gd424@eohsi.rutgers.edu
md1827@eohsi.rutgers.edu
Mandeep.Kaur@ct.gov
gbe4@njit.edu
sa2582@njit.edu
nubia.zuverza@ct.gov
omowunmi.sadik@njit.edu
jason.white@ct.gov
philip.demokritou@rutgers.edu

[1] Nanoscience and Advanced Materials Center, Environmental and Occupational Health Sciences Institute (EOHSI), Rutgers Biomedical Health Sciences, Rutgers University, Piscataway, NJ 08854, USA

[2] Department of Analytical Chemistry, The Connecticut Agricultural Experiment Station, New Haven, CT 06511, USA

[3] Department of Chemistry and Environmental Sciences, 161 Warren Street, New Jersey Institute of Technology, University Heights, Newark, NJ 07102, United States.

*Correspondence: philip.demokritou@rutgers.edu; Tel.: +1-848-445-0155

<u>Supporting Information 1:</u> Justification for the selection of target oral concentrations (TOCs) of PVC MNPs and EPs:

Recent quantitative studies of MNPs in foods reported total plastics concentrations in milk, rice, pork, sardines, and ground beef of 10, 300, 700, 3000, and >10000 µg/g, respectively [1]. Although MNP concentrations in surface, ground, and drinking water were found to be much lower (<1 µg/mL) [2], since food makes up roughly one-fourth of total human intake, average MNP intake could be as high as several hundred µg/mL. We therefore employed relatively conservative target oral concentrations (TOCs) (environmentally relevant food/water concentrations) of 200 and 600 µg/mL. Although these exposure levels are likely higher than those that would be experienced by the average person today, they could be experienced today by someone who consumes large amounts of pork and sardines containing MNPs at the concentrations noted above, and with the expected continued increases in plastics production and pollution, and inevitable corresponding increase in MNP contamination of the environment and food web, they could be experienced by anyone at some point in the near future. In order to account for a 12-fold dilution of the final small intestinal digesta in media prior to exposing SIE cells (required to provide adequate nutrients and avoid direct toxicity from the digesta components [3]), the starting food model (water) concentrations were increased by 12 fold over the TOCs to the adjusted food model concentrations (AFMCs) of 2400 and 7200 µg/mL. Because the starting food model concentrations are also diluted 12-fold during the 3-phase digestion process (which roughly represents the physiological dilution from the mouth to the small intestine), the final concentrations of MNPs applied to the SIE cells were therefore 200/12 = 16.5 µg/mL and 600/12 = 50 µg/mL.

As, Cr, and Pb are among the most widespread and hazardous inorganic contaminants in ecosystems and drinking water, and are commonly used as indicators of toxic-element pollution in environmental studies [4]. PFOS is a well-studied PFAS with strong bioaccumulation potential and documented toxicity in humans and wildlife [5]. Boscalid is a commonly used agricultural fungicide that persists in soil and water. Pesticides have high environmental relevance due to their widespread use, persistence, and impacts on non-target organisms [6]. The TOCs employed for EPs, selected based on existing regulatory limits and reported levels of contamination, were 100 ppb for each of the toxic elements (As(V), Pb(II), and Cr(III)), 50 ppb for PFOS, and 350 ppb for boscalid. Although the Maximum Contaminant Level (MCL) for Pb, As, and Cr established by the US EPA and WHO are 0, 10, and 100 ppb, respectively, significantly higher levels of these toxic elements have been found in water [7]. Similarly, the MCLs for boscalid and PFOS, established by the EPA, are 1.3 ppb and 4 ppt, respectively; however, studies have found significantly higher levels of these organic pollutants in water [8]. As with the MNPs, because of the 12-fold dilution of the final digesta, the AFMCs for toxic elements, PFOS, and boscalid were increased 12-fold from their respective TOCs to 1200, 600, and 4200 ppb, respectively; and because of the overall 144-fold dilution from AFMC to exposure suspensions, the final concentrations of toxic elements, PFOS, and boscalid applied to the SIE cells were 8.3, 4.1, and 29.1 ppb, respectively.

Supporting information 2: Quantification of toxic elements using ICP-MS and organic pollutants using LC-MS

For toxic element analysis, the samples were acidified using an HNO_3 solution before analysis. Briefly, 0.3 mL of a 70% HNO3 solution was added to 4.7 mL of the sample in a 15 mL

polypropylene tube, which was then kept undisturbed for 1 h. The sample was later centrifuged at 3000 rpm for 15 min. The supernatant was filtered using 0.22-micron cellulose filters and used for the determination of toxic elements by ICP-MS. Analysis was performed using an Agilent 7850x ICP-MS to quantify As, Cr, and Pb. For QC purposes, a continuing calibration verification (CCV) sample with a known concentration was run between batches of 15 experimental samples, yielding a recovery of 99-104%. Blank (5% HNO_3), Neodymium (Nd), and Samarium (Sm) were also run to check the precision of trace toxic element detection. A multi-element internal standard (ISTD) solution containing 1% HNO_3/1% HCl + 4% ISO (^{72}Ge, ^{103}Rh, ^{193}Ir, ^{209}Bi), which together cover the full mass range, was included as an internal standard. A one-minute wash with a rinse solution (5 % HCl + 5% HNO_3 + 2 ppm Au) was performed before and after each sample read.

For PFOS and boscalid analysis, 750 µL of sample was taken in a cellulose 0.2 µm centrifuge filter, centrifuged at 14000 rpm for 5 min. The filtered extract was transferred to a polypropylene autosampler with a polyethylene cap and analyzed for PFOS in a 1290 ultra-performance liquid chromatograph (Agilent) coupled with a SciEx 7500 triple-quadrupole mass spectrometer. For QC purposes, a solvent blank (MeOH) and a quality control (QC) sample with a known concentration were run in between batches of 15 experimental samples. Prior to analysis, ^{18}C PFOS internal standard at 10 ng/mL was added to each sample. For QC purposes during boscalid analysis, a solvent blank (Acetonitrile) and a quality control (QC) sample with a known concentration were run between batches of 15 experimental samples. Prior to analysis, 200 ppb of triphenyl phosphate (TPP) internal standard was added to each sample.

At the end of 24 h exposure, 2 mL of apical and basolateral fluid samples from each transwell were collected in sterile amber glass tubes. To determine the concentrations of EPs to which the SIE was exposed (i.e., the starting concentrations in the transwell apical chambers), 2

mL samples of the final small intestinal digesta of water alone (blank digesta), EPs, MNPs, and MNP+EP diluted 1:11 in DMEM media were collected. Toxic elements and organic pollutants were then quantified as detailed above using ICP-MS and LC-MS, respectively.

To obtain an accurate estimation of the percentage loss of EPs across the simulated digestion stages, including losses during pipetting, laboratory consumable usage, and through filter tubes, we conducted a pre-liminary study where we subjected a solution of EPs in water at known concentrations to simulated digestion, acidified and filtered the resulting final small intestinal digesta using cellulose filter tubes, and quantified the toxic elements and organic pollutants in the filtrate using ICP-MS and LC-MS, respectively. The results of our study revealed notable losses during the simulated digestion phases, with Cr(III), As(V), Pb(II), boscalid, and PFOS exhibiting reductions of 4.1%, 1.9%, 6.6%, 6.8%, and 5.2%, respectively **(Supporting Figure 1)**. The percentage loss of EPs was calculated using the following equation (1):

$$\% \, EP \, loss = 100 \times \frac{Known \, EP \, concentratoin - Filtrate \, EP \, concentration}{Known \, EP \, concentration} \quad (1)$$

To estimate the percentage loss of EPs in the transwell SIE model over a 24-hour period for translocation assessment, we conducted a similar preliminary study. In this study, we suspended a known concentration of EPs in water and applied it to a sterile transwell plate. The plate was then incubated for 24 h. After incubation, basolateral fluid was collected, acidified, and filtered through cellulose filter tubes. The filtrates were then analyzed for toxic elements and organic pollutants using ICP-MS and LC-MS, respectively. The percentage loss of EPs was calculated using Equation (1). Across the stages of toxicity and translocation assessment in the transwell model, we noted further losses of 2.1%, 2.7%, 7.1%, 5.2%, and 4.8% for Cr(III), As(V), Pb(II), boscalid, and PFOS, respectively **(Supporting Figure 1)**.

Supporting information 3: In-vitro simulated digestion and preparation of *in vitro* triculture SIE model

A three-phase (oral, gastric, and small intestinal) *in vitro* simulated digestion was conducted as detailed previously [9, 10, 11]. Briefly, in the oral phase, blank digesta (CGW), EP mixture alone, PVC-UA, PVC-A, PVC-I, EP-PVC-UA, EP-PVC-A, and EP-PVC-I in CGW were mixed 1:1 with pre-warmed (37 ˚C) simulated saliva containing mucin and other salts at a pH of 6.8 in amber glass tubes. The tubes were inverted manually for 15 sec to complete the oral phase. To simulate the gastric phase of digestion, the resultant oral phase digesta was combined at a 1:1 ratio with pre-warmed simulated gastric fluid, comprised of pepsin, hydrochloric acid (HCl), and sodium chloride (NaCl), and incubated in an orbital shaker at 200 rpm for 2 h. The gastric digesta was then mixed with bile salts, pancreatin (which includes the complete set of pancreatic digestive enzymes), and additional salts, resulting in a dilution of the gastric digesta by a factor of three. The pH of this mixture was adjusted to 7.0 by adding sodium hydroxide (NaOH) to replicate small intestinal fluid. The SI digesta was then incubated in the orbital shaker at 37 °C and 200 rpm for 2 h to complete the small intestinal phase.

The transwell triculture SIE model was prepared as detailed previously [10, 11]. Briefly, Caco-2 (Sigma-Aldrich, MO, USA) (a cell line derived from human colorectal adenocarcinoma, which resembles intestinal enterocytes upon maturation in culture) and HT29-MTX (Sigma-Aldrich, MO, USA) (mucus-secreting goblet cells) cells were cultured in 150 cm^2 cell culture flasks (Corning, NY, USA) in high-glucose DMEM media (Thermo Fisher Scientific, MA, USA) supplemented with 10% heat inactivated fetal bovine serum (HI-FBS) (Sigma-Aldrich, MO, USA), a 10 mM HEPES buffer (Lonza, Basel, Switzerland), 100 IU/mL penicillin plus 100 µg/mL streptomycin (Corning,

NY, USA), and non-essential amino acids (1/100 dilution of 100X solution) (Thermo Fisher Scientific, MA, USA). Raji-B cells (Sigma-Aldrich, MO, USA) were cultured in RPMI 1640 media (Thermo Fisher Scientific, MA, USA) supplemented with 10% HI-FBS, 10 mM HEPES buffer, and 100 IU/mL penicillin plus 100 µg/mL streptomycin. Caco-2 and HT29-MTX cells were collected at passages 10–25 using TrypLE Express (Thermo Fisher Scientific, MA, USA) and then resuspended in complete DMEM media at a 3×10^5 live cells/mL density for each cell type. These two cell suspensions were combined in a ratio of 3 parts Caco-2 cells to 1-part HT29-MTX cells. A 1.5 mL portion of the cell mixture was then added to the upper (apical) compartments of 6-well transwell plates (24 mm, polycarbonate membrane with an 8 µm pore size) (Corning, NY, USA), while 2.5 mL of complete DMEM was introduced into the lower (basolateral) compartments. The transwell plates were incubated at 37 °C and 5% CO_2, with media changes occurring after 4 days and subsequently every other day until day 16. The media in the lower compartments were replaced on days 16 and 17 with 2.5 mL of a Raji-B cell suspension, which was harvested at passages 10-20 at a concentration of 1×10^6 live cells/mL in a 1:1 mixture of complete DMEM and complete RPMI media.

The exposure of the SIE model to the SI digesta of CGW (blank digesta), EP mixture alone, PVC-UA, PVC-A, PVC-I, EP-PVC-UA, EP-PVC-A, and EP-PVC-I in CGW, was performed on day 18 of SIE culture [10]. The small intestinal digesta of each sample were mixed at a 1:11 ratio with high-glucose DMEM media without phenol red, supplemented with 10 mM HEPES buffer, 100 IU/mL penicillin, 100 µg/mL streptomycin, and non-essential amino acids. The apical media was replaced with 2.5 mL of either media alone (negative control or NC) or digesta-media mixtures, and the basolateral media was replaced with 2.5 mL of complete DMEM without phenol red, supplemented with 10% HI-FBS, 10 mM HEPES buffer, 100 IU/mL penicillin, 100 µg/mL

streptomycin, and non-essential amino acids. The transwell plates were then incubated at 37 °C and 5% CO_2 for 24 h.

Supporting information 4: Toxicological analysis

The ROS production (oxidative stress) was assessed using the OxiSelect *in vitro* assay kit (Cell Biolabs, CA, USA) according to the manufacturer's protocol, with minor adjustments [10]. The 1X Catalyst and DCFH solution (final reaction mixture) was prepared as guided by the manufacturer. After 6 h of the 24-h incubation, 150 µL of apical fluids were collected in sterile Eppendorf tubes from the transwell assigned to the negative control (NC), CGW (blank digesta), EPs, PVC MNPs, and PVC MNP-EPs. The tubes were centrifuged at 5000 × g for 5 min. To prepare standards, a 2-five-fold serial dilution in sterile water was performed with 20 µM H_2O_2 solution. Further, 50 µL of NC, blank digesta, EPs, PVC MNPs, PVC MNP-EPs, and H_2O_2 standards were dispensed into a black-walled, clear-bottom 96-well plate. A 50 µL of the catalyst solution was added to each well, and the plate was incubated for 5 min at RT. A 100 µL of the final reaction mixture was added to each well, and the plate was incubated for 30 min at RT. Fluorescence was measured at 480/530 nm (excitation/emission) using a SpectraMax M-5 (Molecular Devices, CA, USA) microplate reader, and equivalent µM H_2O_2 concentrations in the samples were determined from a standard curve generated from H_2O_2 standards. The data were represented by fold change vs. NC.

The release of LDH was measured using a Pierce LDH assay kit (Sigma-Aldrich, St., MO, USA) following the manufacturer's guidelines with certain modifications [10]. Briefly, 150 µL of apical media from one of the two plates assigned to the NC was replaced with 150 µL of 2X RIPA buffer (Thermo Fisher Scientific, MA, USA), 45 min before the end of the 24-h exposure period to

create a positive control (PC). After the 24-h exposure, 150 μL of apical fluid was collected in sterile Eppendorf tubes from each transwell designated for PC, NC, blank digesta, EPs, MNPs, and MNP-EPs and centrifuged at 10,000 × g for 5 min. A 50 μL sample from centrifuged apical fluids was dispensed in a black-walled, clear-bottom 96-well plate (BD Biosciences, NJ, USA), where a 50 μL reaction mixture, prepared according to the manufacturer's instructions, was added. The plate was incubated in the dark at RT for 30 min. Further, 50 μL of stop solution was added to each well, and absorbance was measured at 680 (A680) and 490 (A490) nm using a microplate reader. Background corrected absorbance, A, was calculated for each well by subtracting A_{680} from A_{490}. The percentage cytotoxicity for each treatment well was calculated using equation (2):

$$\% \, Cytotoxicity = 100 \times \frac{A_T - A_{NC}}{A_{PC} - A_{NC}} \qquad (2)$$

where A_T is the absorbance of the treatments (blank digesta, EPs, PVC MNPs, and PVC MNP-EPs), A_{NC} is the absorbance of the negative control, and A_{PC} is the absorbance of the positive control.

Trans-epithelial electrical resistance (TEER) was evaluated using the EVOM2 Epithelial V/Ω Meter with a chopstick Electrode Set (World Precision Instruments, FL, USA) as detailed earlier [10]. After LDH analysis, the apical and basolateral fluids were replaced with 3 mL PBS. TEER measurements were taken in PC, NC, blank digesta, EPs, MNPs, and MNP-EPs to evaluate their impact on epithelial barriers and tight junction integrity. The TEER values were expressed in Ω/cm^2.

The dextran permeability was assessed using two fluorescently labeled dextrans, Alexa Fluor 488 3 kDa and Texas Red 70 kDa (Thermo Fisher Scientific, MA, USA) [10]. A 1 mL working solution per transwell insert to be tested, comprising a mixture of 25 μg/mL of AF488 3 kDa dextran and 50 μg/mL of Texas 70 kDa dextran, was prepared in PBS. After TEER analysis, the transwells were washed twice with 3 mL PBS. Subsequently, 1 mL of working dextran solution was

added to the apical and 2 mL of PBS to the basolateral compartments. The transwell plates were incubated at 37 °C and 5% CO_2 for 1 h. A 200 µL of basolateral fluids from plates assigned to PC, NC, blank digesta, EPs, MNPs, and MNP-EPs were collected and placed in a black-walled, clear-bottom 96-well plate. Fluorescence was measured at Ex 495 nm/Em 519 nm for Alexa Fluor 488 3 kDa dextran and Ex 595 nm/615 nm for Texas Red 70 kDa dextran using a microplate reader. Apparent permeability, P_{app} (cm/s), was calculated using equation (3):

$$P_{app} = \frac{dQ}{dt \times A \times C_0} \qquad (3)$$

where, dQ represents the quantity of dextran (in µg) present in the basolateral compartment, which is determined through fluorescence measurements and standard curves specific to each dextran; the variable dt indicates the duration (in seconds) from the moment the dextran is introduced into the apical compartment until it is measured in the basolateral compartment; A denotes the surface area of the transwell; and C_0 refers to the initial concentration of dextran in the apical compartment.

Supporting information 5: Sample collection and quantification of MNP uptake and translocation in the SIE using Py-GC-MS

At the end of 24 h of exposure, 2 mL of apical and basolateral fluid samples from each transwell were collected for MNP analysis. Finally, following assessment of dextran permeability, apical and basolateral compartments were washed with 4 mL of PBS, and 0.5 mL of 2X RIPA buffer (Thermo Fisher Scientific, MA, USA) was added to each apical chamber and incubated at RT for 10 min. Two mL of sterile DI water was then added to each apical compartment and pipetted up and down, and a cell scraper was used to remove all cell debris from the insert membranes. The cell lysates were then collected in sterile amber glass tubes. To determine the concentrations of

MNPs to which the SIE was exposed, 2 mL samples of the final small intestinal digesta of water alone (blank digesta), EPs, MNPs, and MNP+EP diluted 1:11 in DMEM media (as described above) were collected in sterile amber glass tubes for MNP analysis.

The translocation and uptake of MNPs were quantified using Py-GC-MS. For that, 500 µL of the samples were preheated in a water bath at 55 °C for an hour. Subsequently, 100 µL of 10% potassium hydroxide solution was added to the preheated samples at a 5:1 (v/v) ratio. The resultant mixture was incubated at 55 ˚C. The progress of KOH digestion on the cell lysate was monitored at a timely interval. Py-GC/MS analysis of the PVC MNPs in cell lysates, apical and basolateral compartments, was performed on a pyrolyzer unit (Pyroprobe 6150, CDS) connected to an Agilent gas chromatograph-mass spectrometer. (Agilent 8890 GC system.) The Pyrolyzer is interfaced with the GC/MS instrument via a split/splitless injection port. The GC injection port is linked to the triple quadrupole mass detector. The pyrolysis of the PVC sample was carried out at 600 °C for 20 s with 300 °C as the interface temperature. The pyrolysis product was automatically injected into the split mode with a ratio of 50:1. The samples were initially subjected to pyrolysis at 600 °C for 20 seconds using helium gas. The PVC fragments from pyrolysis were separated using a non-polar GC column (DB-5, 30 m × 0.25 mm × id, 0.25 µm) and an oven ramp (initial temperature of 50 ◦C maintained during 1 min, raised at 3 ◦C/min until 325 ˚C, the final temperature, maintained during 10◦C/ min), 325 ˚C for 5 min. The mass analysis was performed in SCAN and electron impact modes. The analysis of PVC MNPs underwent ionization, and the resultant fragments were analyzed by a triple quadrupole mass spectrometer operating at 2 scans/s over the range of 35 to 350 m/z. The specific parameters for the single-shot pyrolysis-GC/MS measurements for PVC MNPs are provided in **Supporting Table 2**.

Supporting information 6: Assessment of effects on gene expression (RNA-seq)

The samples for RNA sequencing (RNA-seq) expression analysis were collected as previously detailed by our group [12]. Briefly, after 24 h exposure, the basolateral fluid was removed, and the apical fluid was replaced with 500 µL of TRIzol® reagent. The cell lysates were collected in sterile Eppendorf tubes and stored at −80 °C prior to RNA isolation and analysis. RNA samples were sent to Novogene (Sacramento, CA) for RNA extraction, cleanup, and single-end sequencing at 6 Gb per sample. Raw sequencing data (fastq) were first quality checked using fastQC (v0.11.3) and were further aligned to human (hg19) genomes using kallisto (v0.50.0) [13] to generate H5 files, and differential expression analysis was performed using DESeq2 (v1.46.0) [14] in R(v4.4). Significantly differentially expressed genes are defined as average FPKM > 1 and p-adjusted < 0.05.

Supporting information 7: Physicochemical properties of PVC MNPs across their lifecycle

PVC-A MNPs generated by cryomilling were UV-aged for 21 days, using a Q-Sun Xenon test chamber, replicating approximately 180 days of outdoor aging [15]. MALD analysis revealed a D_{90} value of 10.7 µm for both PVC-UA and PVC-A, indicating that 90% of PVC-UA and PVC-A particles were smaller than 10.7 µm [15]. Scanning electron microscopy (SEM) images showed that PVC-UA and PVC-A MNPs exhibited irregular morphologies with sharp edges, and had mean particle diameters of 5.5 ± 2.2 µm and 2.7 ± 1.4 µm, respectively **(Supporting Figure 2A-B)** [15]. The zeta potentials of PVC-UA and PVC-A in water, determined by electrophoretic light scattering (ELS) analysis, were both strongly negative, at −26.0 ± 0.8 mV and −33.0 ± 0.8 mV, respectively [15]. Diffuse reflectance infrared Fourier transform spectroscopy (DRIFT) analysis revealed oxygen-containing functional groups in PVC-A MNPs **(Supporting Figure 3)**, while X-ray photoelectron

spectroscopy (XPS) showed an increase in oxygen content of 5.5% compared to PVC-UA, indicating photo-oxidation as a result of UV-aging for 21 days **(Supporting Figure 4 and Supporting Tables 3 and 4)** [15].

The PVC-I MNPs used in this study were generated using the integrated exposure generation *system (*INEXS), which enables the collection and size fractionation of particles produced during the thermal decomposition/incineration of test materials [15]. Dynamic light scattering (DLS) and ELS analysis revealed that PVC-I particles suspended in water had an average hydrodynamic diameter, d_H, of 220 ± 2.8 nm, and a zeta potential of -34.1 ± 0.2 mV. Elemental and organic carbon analysis revealed that PVC-I was composed primarily of organic carbon (99.71%), with relatively small amounts of elemental carbon (0.29%) [15]. In addition, gas chromatography-mass spectrometry (GC-MS) analysis of PVC-I MNPs revealed that the particles contained high amounts (43.16 µg/kg) of polycyclic aromatic hydrocarbons (PAHs) **(Supporting Figure 5)**. These PAHs included low-molecular-weight compounds such as phenanthrene and acenaphthene, which are generally less toxic, and high-molecular-weight compounds such as chrysene, pyrene, benzo[*a*]anthracene, benzo[b]fluoranthene, and benzo[*a*]pyrene, which are known carcinogens [15].

It is worth noting that the three MNPs generated throughout the life cycle of PVC plastic, as described above, have distinct and unique physicochemical properties. These properties are anticipated to impact both their interactions with EPs and, more importantly, the toxicity and bioavailability of EPs and MNPs.

Supporting Tables

**Sample**	**<u>PFOS</u> TOC: 50 ppb**	**<u>Boscalid</u> TOC: 350 ppb**	**<u>Cr(III)</u> TOC: 100 ppb**	**<u>As(V)</u> TOC: 100 ppb**	**<u>Pb(II)</u> TOC: 100 ppb**	**<u>MNP</u> (ppm)**
CGW (blank)	0	0	0	0	0	0
MNP (TOC: 200 ppm)	0	0	0	0	0	2400
MNP (TOC: 600 ppm)	0	0	0	0	0	7200
EPs	600	4200	1200	1200	1200	0
EP-MNP (TOC: 200 ppm)	600	4200	1200	1200	1200	2400
EP-MNP (TOC 600 ppm)	600	4200	1200	1200	1200	7200

Supporting Table 1. Starting adjusted food model (CGW) concentrations (AFMCs) for MNPs and EPs.

Apparatus	Parameters	Settings
**Pyrolyzer**	Carrier gas	Helium
	Pyrolysis temperature	600 °C
	Interface temperature	300 °C
	Pyrolysis time	20 seconds
**Gas Chromatography**	Column	HP-5MS (15m x 250 µm x 0.25 µm)
	Injector port temp	320 °C
	Column oven temp	50 °C (2 min)
	Injector mode	320 °C (20 °C /min, 14 min)
	Split Ratio	Split (5:1)
**Mass Spectrometer**	Ion source temperature	230 °C
	Ionization energy	Electron ionization (EI): 70 eV
	Scan range	35 to 350 m/z

Supporting Table 2. Parameters for Single-shot Pyrolysis-GC/MS measurements.

Sample	C (%)	Cl (%)	O (%)	Other (%)
**PVC-UA**	73.1	20.7	5.6	0.6
**PVC-A**	66.5	20.5	11.1	1.9

Supporting Table 3: The elemental composition on the surface of un-aged (PVC-UA) and UV-aged (PVC-A) PVC MNPs. The table has been adapted from data published in an open-access article by Das *et al.* [15]. Available under a CC-BY 4.0. Copyright 2025 Elsevier B.V.

	C1s			Cl2p	O1s		
**Sample**	**284.6 eV C-C/C-H**	**286.1 eV C-Cl/C-O**	**288.4 eV C=O**	**Cl (%)**	**532.2 eV C-O**	**534.6 eV C=O**	**Other (%)**
**PVC-UA**	36.99	32.63	3.50	20.73	4.89	0.68	0.58
**PVC-A**	32.67	28.56	5.26	20.5	9.99	1.07	1.97

Supporting Table 4. Summary of peak assignments with atomic percentage. The table has been adapted from data published in an open-access article by Das *et al.* [15]. Available under a CC-BY 4.0. Copyright 2025 Elsevier B.V.

	PVC-UA (% sorption)	PVC-A (% sorption)	PVC-I (% sorption)	Statistical comparison
**Cr**	18.6±4.2	21.7±1.9	9.5±2.0	PVC-A ≈ PVC-UA (ns); PVC-A > PVC-I ($p < 0.001$)
**As**	20.0±1.4	27.2±0.9	15.0±1.4	PVC-A > PVC-UA ($p < 0.01$); PVC-A > PVC-I ($p < 0.001$)
**Pb**	22.8±1.2	29.9±1.1	24.1±1.6	PVC-A > PVC-UA ($p < 0.01$); PVC-A > PVC-I ($p < 0.01$)
**Boscalid**	38.3±1.3	37.8±0.7	41.9±3.0	No significant differences among PVC MNPs (ns)
**PFOS**	20.6±3.7	23.0±4.3	26.6±3.9	No significant differences among PVC MNPs (ns)

Supporting Table 5. Sorption (%) of EPs by PVC-UA, PVC-A, and PVC-I in water.

		PVC-UA (% sorption)	PVC-A (% sorption)	PVC-I (% sorption)	Statistical comparison
**Oral phase**	**Cr**	18.5±4.2	22.41±0.6	10.1±0.9	PVC-UA > PVC-I ($p < 0.01$); PVC-A > PVC-I ($p < 0.001$); PVC-UA ≈ PVC-A (ns)
	**As**	18.7±3.2	25.0±1.3	16.6±1.2	PVC-A > PVC-UA ($p < 0.05$); PVC-A > PVC-I ($p < 0.05$); PVC-UA ≈ PVC-I (ns)
	**Pb**	13.6±1.1	10.8±4.3	13.7±2.3	No significant differences among PVC MNPs (ns)
	**Boscalid**	20.0±2.5	24.4±2.6	26.6±2.0	No significant differences among PVC MNPs (ns)
	**PFOS**	18.3±2.2	23.3±3.4	23.6±6.0	No significant differences among PVC MNPs (ns)
**Gastric phase**		**PVC-UA (% sorption)**	**PVC-A (% sorption)**	**PVC-I (% sorption)**	**Statistical comparison**
	**Cr**	12.5±1.7	17.6±1.9	9.7±0.5	PVC-A > PVC-I ($p < 0.05$); PVC-UA ≈ PVC-A (ns); PVC-UA ≈ PVC-I (ns)
	**As**	11.7±0.7	16.3±1.5	16.3±0.3	PVC-A > PVC-UA ($p < 0.05$); PVC-I > PVC-UA ($p < 0.05$); PVC-A ≈ PVC-I (ns)
	**Pb**	10.8±1.0	14.4±0.4	11.8±0.4	No significant differences among PVC MNPs (ns)
	**Boscalid**	10.4±1.9	21.9±2.2	22.3±0.4	PVC-A > PVC-UA ($p < 0.001$); PVC-I > PVC-UA ($p < 0.001$); PVC-A ≈ PVC-I (ns)

	**PFOS**	16.6±7.6	10.5±2.8	19.4±5.8	No significant differences among PVC MNPs (ns)
**Small intestinal phase**		**PVC-UA (% sorption)**	**PVC-A (% sorption)**	**PVC-I (% sorption)**	**Statistical comparison**
	**Cr**	9.0±0.5	14.7±1.8	8.8±0.8	PVC-A > PVC-UA ($p < 0.01$); PVC-A > PVC-I ($p < 0.01$); PVC-UA ≈ PVC-I (ns)
	**As**	4.1±1.0	10.4±1.6	11.2±0.5	PVC-A > PVC-UA ($p < 0.01$); PVC-I > PVC-UA ($p < 0.01$); PVC-A ≈ PVC-I (ns)
	**Pb**	8.8±2.4	12.1±0.5	4.4±1.2	PVC-A > PVC-UA ($p < 0.05$); PVC-A > PVC-I ($p < 0.001$); PVC-UA > PVC-I ($p < 0.01$)
	**Boscalid**	3.9±1.9	13.9±2.4	8.5±2.2	PVC-A > PVC-UA ($p < 0.001$); PVC-A > PVC-I ($p < 0.05$); PVC-UA ≈ PVC-I (ns)
	**PFOS**	5.0±1.5	9.2±3.2	8.3±2.9	No significant differences among PVC MNPs (ns)

Supporting Table 6. Sorption (%) of EPs by PVC-UA, PVC-A, and PVC-I across the GIT.

Sample Name	Particle Size distribution from MALD		
	D_{10} (µm)	D_{50} (µm)	D_{90} (µm)
PVC-UA_CGW	2.36±0.007	5.01±0.018	8.78±0.058
EP-PVC-UA_CGW	2.33±0.009	5.09±0.029	9.05±0.103
PVC-A_CGW	2.47±0.002	4.37±0.022	7.11±0.051
EP-PVC-A_CGW	2.46±0.016	4.87±0.046	8.47±0.169
PVC-I_CGW	0.0557±0.0001	0.176±0.003	0.575±0.005
EP-PVC-I_CGW	0.0571±0.0003	0.188±0.001	1.24±0.259
PVC-UA_SI	6.73±0.0086	10.9±0.051	16.9±0.180
EP-PVC-UA_SI	7.83±0.032	11.9±0.080	17.8±0.152
PVC-A_SI	2.75±0.043	8.17±0.227	19.8±0.533
EP-PVC-A_SI	9.25±0.093	13.5±0.179	19±0.400
PVC-I_SI	0.808±0.042	3.63±0.095	7.08±0.714
EP-PVC-I_SI	0.713±0.012	4.19±0.422	8.44±0.255

Supporting Table 7. Particle sizes from multi-angle laser diffraction (MALD) analysis.

	EP	EP-PVC-UA (C1)	EP-PVC-UA (C2)	EP-PVC-A (C1)	EP-PVC-A (C2)	EP-PVC-I (C1)	EP-PVC-I (C2)	Statistical comparison
**Cr**	9.0±1.2	8.7±0.03	10.3±1.7	16.7±2.5	16.1±1.9	9.9±0.5	11.8±1.9	EP-PVC-A (C1) > EP ($p < 0.05$); EP-PVC-A (C2) > EP ($p < 0.01$)
**As**	7.1±3.1	8.±0.7	6.9±0.8	9.3±3.2	8.1±1.6	8.7±0.2	9.6±0.1	No significant differences (ns)
**Pb**	11.9±0.4	12.2±0.1	10.5±1.1	12.1±0.8	18.4±3.8	11.6±0.8	12.8±0.07	EP-PVC-A (C2) > EP ($p < 0.01$)
**Boscalid**	11.3±0.8	11.6±1.0	11.3±1.3	10.8±0.3	14.0±0.7	14.6±1.3	15.1±1.5	EP-PVC-A (C2) > EP ($p < 0.05$); EP-PVC-I (C1) > EP ($p < 0.05$); EP-PVC-I (C2) > EP ($p < 0.05$)
**PFOS**	9.8±1.6	8.9±0.9	8.7±0.9	10.5±1.6	15.4±0.2	8.7±0.7	14.9±0.3	EP-PVC-A (C2) > EP ($p < 0.01$); EP-PVC-I (C2) > EP ($p < 0.01$)

Supporting Table 8. Effect of PVC MNPs on the translocation of EPs across SIE.

	% Uptake	Statistical comparison	% Translocation	Statistical comparison
**PVC-UA (C1)**	6.7±1.8	PVC-A (C1) < PVC-I (C1) ($p < 0.05$); PVC-UA (C1) < PVC-I (C1) ($p < 0.05$); EP-PVC-A (C1) < EP-PVC-I (C1) ($p < 0.01$); EP-PVC-UA (C1) < EP-PVC-I (C1) ($p < 0.01$)	4.5±2.2	PVC-A (C1) ≈ EP-PVC-A (C1) (ns); PVC-A (C2) < EP-PVC-A (C2) ($p < 0.01$); PVC-I (C1) ≈ EP-PVC-I (C1) (ns); PVC-A (C2) < EP-PVC-A (C2) ($p < 0.05$); EP-PVC-A (C2) < EP-PVC-I (C2) ($p < 0.05$)
**PVC-UA (C2)**	7.1±2.4		5.3±1.8	
**EP-PVC-UA (C1)**	5.3±1.8		4.8±2.6	
**EP-PVC-UA (C2)**	6.2±2.0		5.7±1.2	
**PVC-A (C1)**	5.6±2.8		5.2±1.6	
**PVC-A (C2)**	8.6±1.4		5.0±1.3	
**EP-PVC-A (C1)**	6.3±0.6		5.9±1.8	
**EP-PVC-A (C2)**	8.7±2.1		10.2±0.5	
**PVC-I (C1)**	11.3±0.3		9.5±0.3	
**PVC-I (C2)**	11.3±1.2		11.3±1.1	
**EP-PVC-I (C1)**	11.5±1.0		9.3±0.1	
**EP-PVC-I (C2)**	10.2±2.8		14.7±0.9	

Supporting Table 9. Effects of EPs on the uptake and translocation of PVC MNPs across SIE.

**Genes**	**EP vs EP-PVC-CW ($\log_2$ fold change)**	***p-value***
Cldn5	-3.6	$p < 0.05$
Notch1	-1.7	$p < 0.05$
Cdh5	+5.3	$p < 0.01$
Gja4	-2.1	$p < 0.05$
Itga4	-1.6	$p < 0.05$
Itgal	-1.1	$p < 0.05$
	**EP vs EP-PVC-I (fold change)**	**p-value**
Cldn11	-4.7	$p < 0.05$
Notch1	-2.4	$p < 0.05$
Cdh5	+4.7	$p < 0.05$
Itgal	-4.4	$p < 0.05$
Itga7	-1.8	$p < 0.05$
Dsg4	-4.9	$p < 0.01$
Icam2	-2.6	$p < 0.05$
	**PVC-CW vs EP-PVC-CW**	**p-value**
Cldn5	-5.0	$p < 0.05$
Cldn11	-4.3	$p < 0.05$
Jam3	-2.4	$p < 0.01$
Cdh4	-1.3	$p < 0.001$
Gja4	-6.3	$p < 0.001$
	**PVC-I vs EP-PVC-I**	**p-value**
Cldn14	-3.8	$p < 0.05$
Cdh6	-4.5	$p < 0.05$
Dsg4	-6.1	$p < 0.001$

Supporting Table 10. Effect of EPs, EP-PVC-A, and EP-PVC-I MNPs on gene expression in SIE.

Supporting Figures

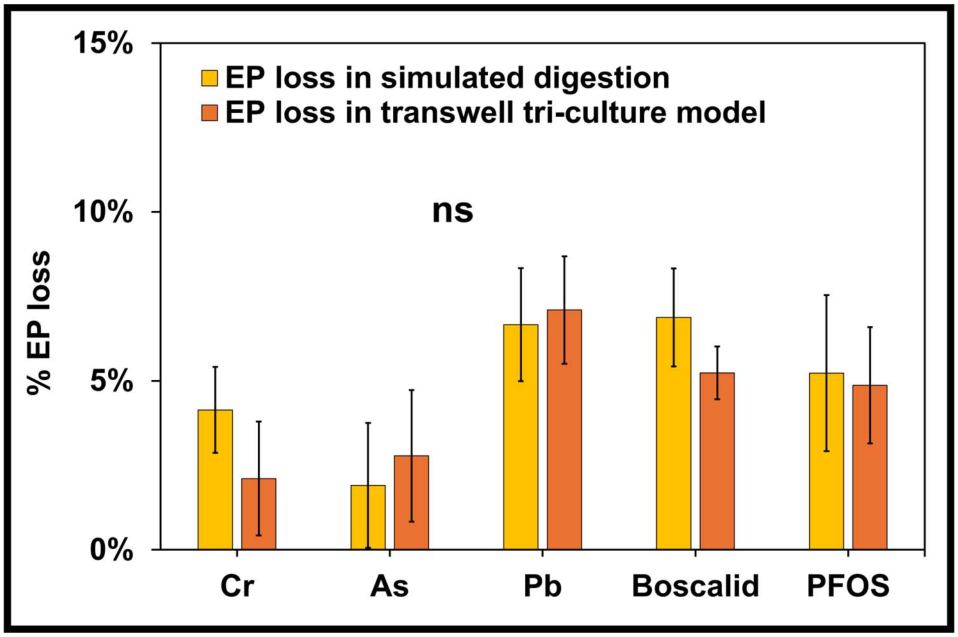

Supporting Figure 1. EP loss % across *in vitro* simulated digestion and translocation assessment stages in the transwell triculture model. Data are shown as mean ± SD, ns=non-significant.

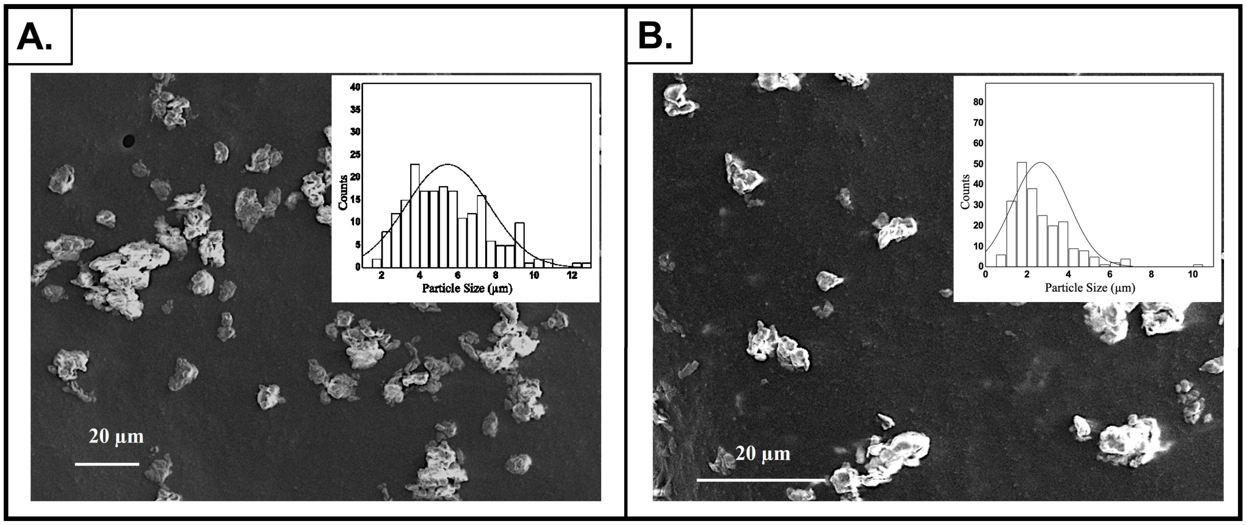

Supporting Figure 2. Effect of UV-aging on the surface morphology and size distribution of PVC MNPs. A. SEM image of un-aged PVC MNPs. **B.** SEM image of 21-day UV-aged PVC MNPs. The figure has been adapted from data published in an open-access article by Das *et al.* [15]. Available under a CC-BY 4.0. Copyright 2025 Elsevier B.V.

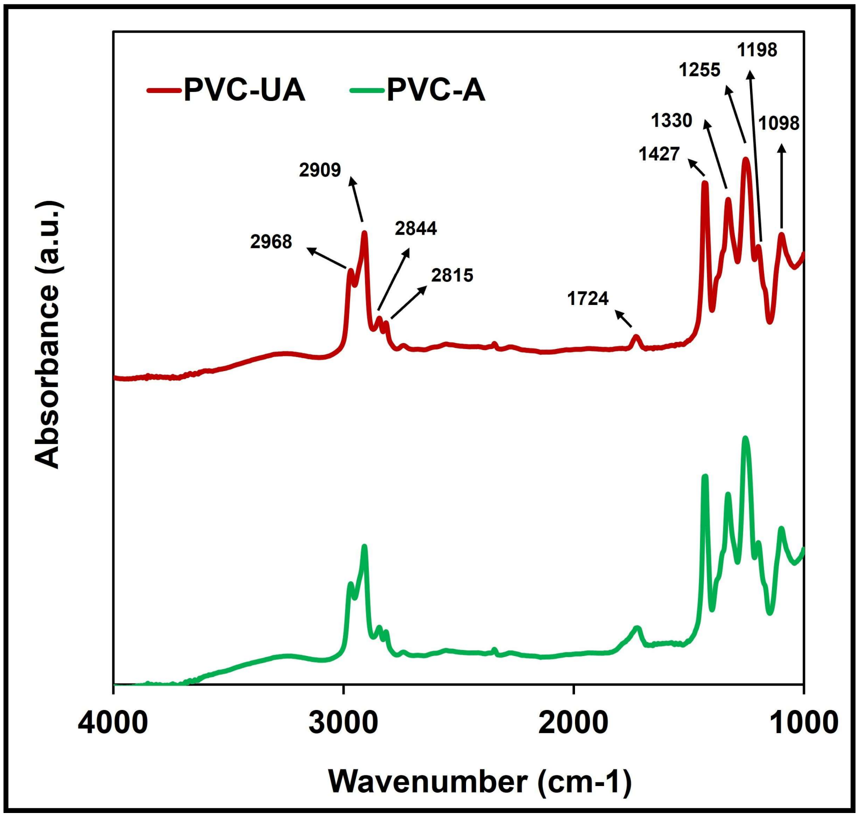

Supporting Figure 3. Diffuse Reflectance Infrared Fourier Transform (DRIFT) spectra of cryomilled and un-aged (PVC-A) and cryomilled and 21-day UV-aged (PVC-A) PVC MNPs. The figure has been adapted from data published in an open-access article by Das *et al.* [15]. Available under a CC-BY 4.0. Copyright 2025 Elsevier B.V.

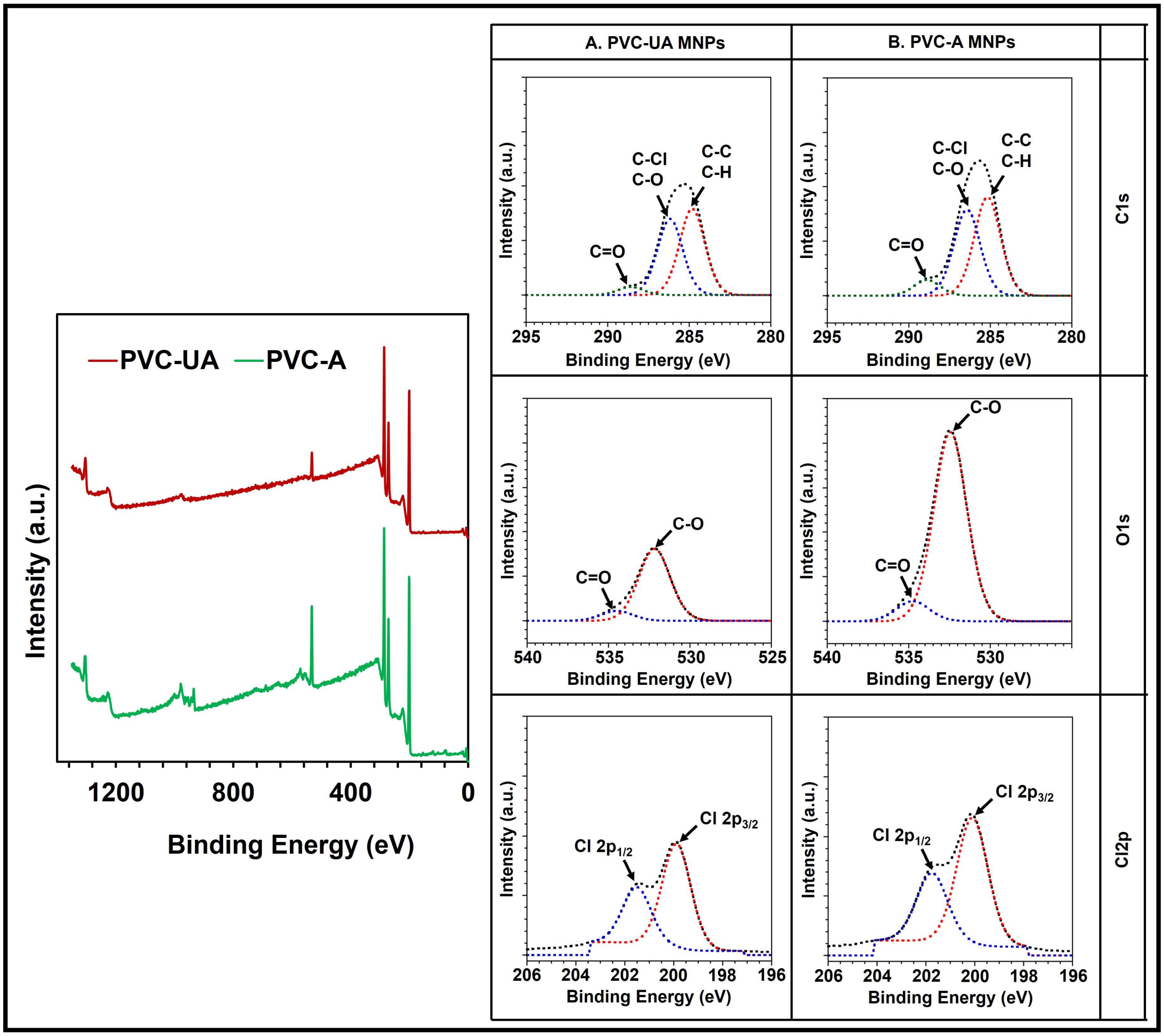

Supporting Figure 4. Effect of UV-aging on the surface elemental composition of PVC MNPs. A. XPS spectra of cryomilled and un-aged (PVC-A) PVC MNPs. **B.** XPS spectra of cryomilled and 21-day UV-aged (PVC-A) PVC MNPs. The figure has been adapted from data published in an open-access article by Das *et al.* [15]. Available under a CC-BY 4.0. Copyright 2025 Elsevier B.V.

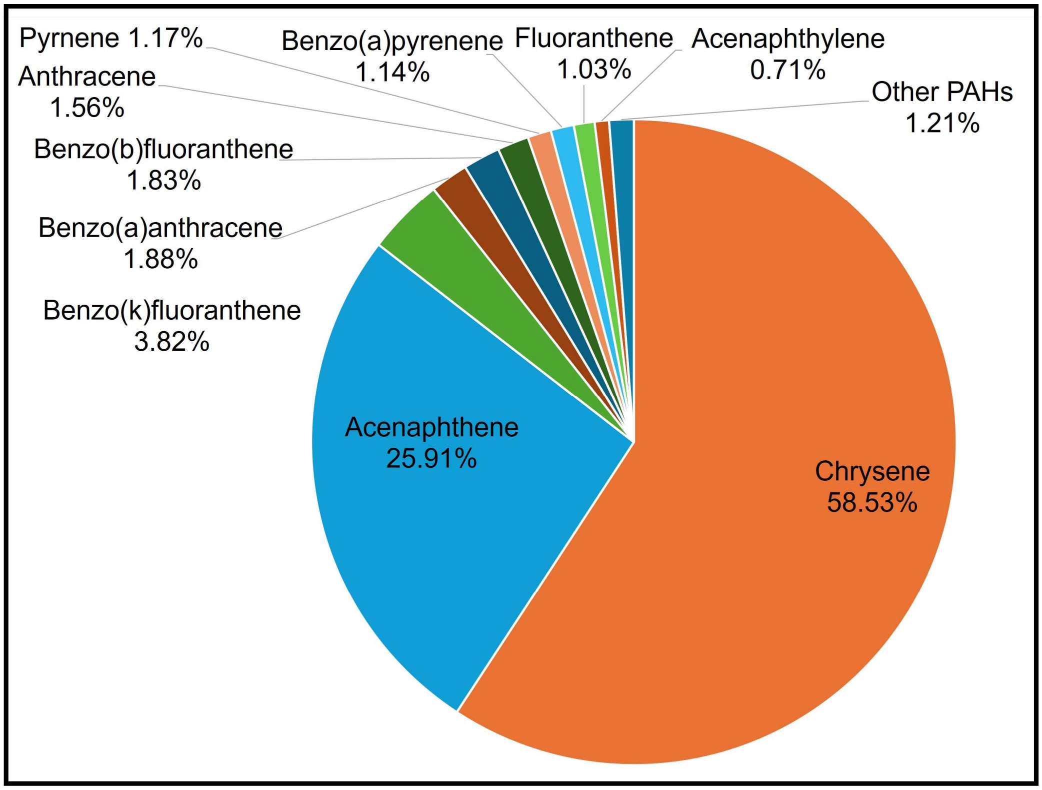

Supporting Figure 5. The polycyclic aromatic hydrocarbons (PAHs) composition of incinerated (PVC-I) PVC MNPs was assessed by gas chromatography (GC) with mass spectrometry. The figure has been adapted from data published in an open-access article by Das *et al.* [15]. Available under a CC-BY 4.0. Copyright 2025 Elsevier B.V.

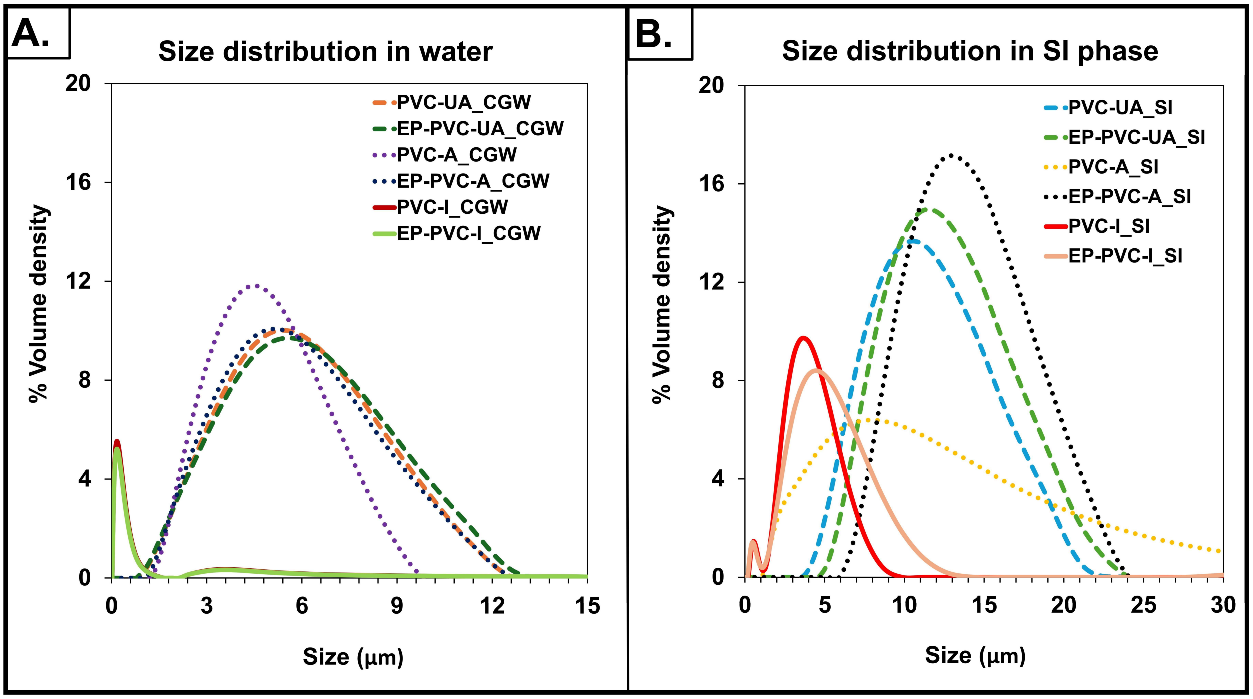

Supporting Figure 6. Volume-weighted size distributions of PVC MNPs and PVC MNP+EP mixtures in water and small-intestinal digesta.

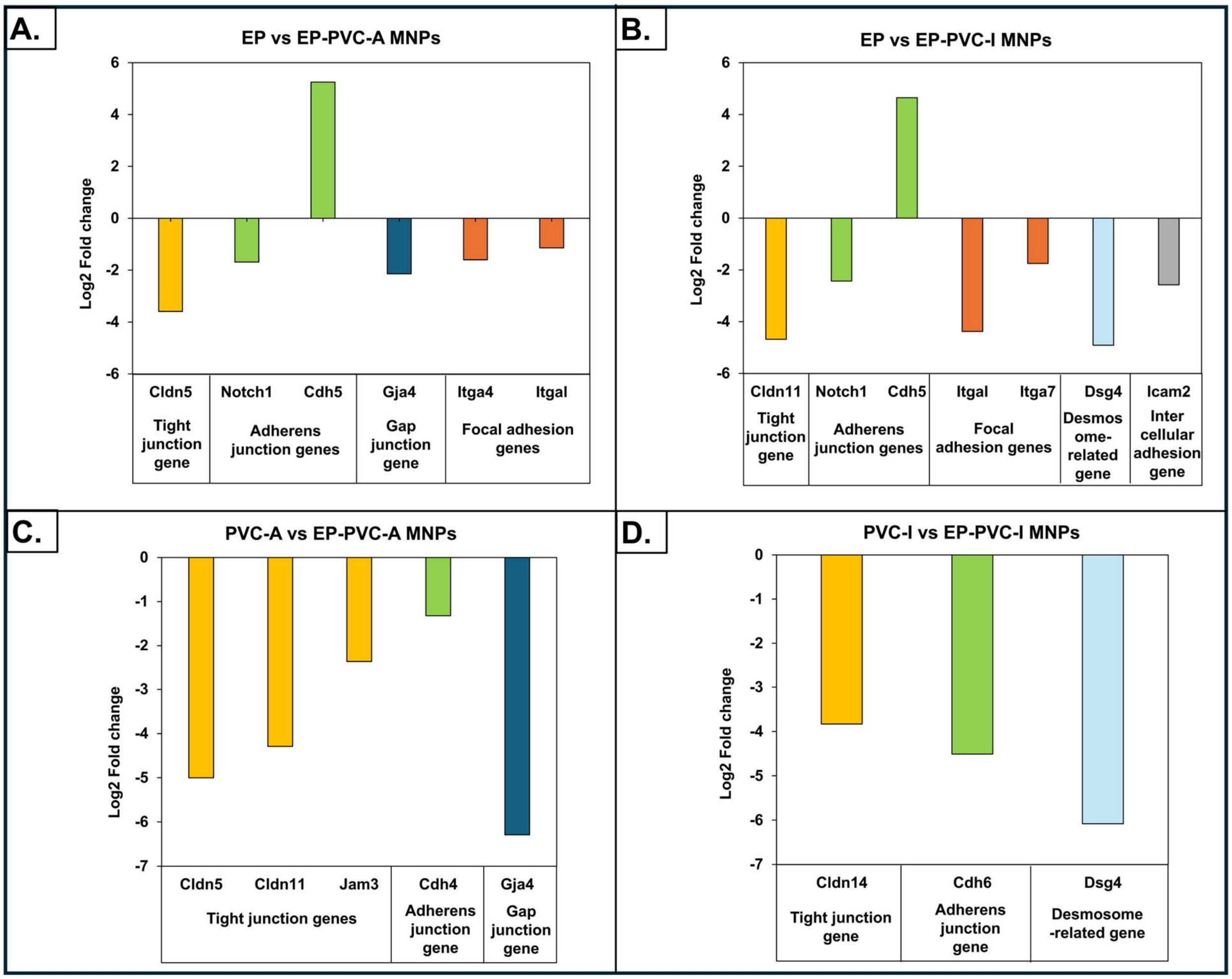

Supporting Figure 7. Effect of EPs, EP-PVC-A, and EP-PVC-I MNPs on gene expression in SIE. A. Influence of PVC-A MNPs on the bioavailability of EPs. **B.** Influence of PVC-I MNPs on the bioavailability of EPs. mRNA expression log_2 fold changes for EP-PVC-A and EP-PVC-I MNPs were calculated compared to the expression levels observed in cells exposed to EPs alone. **C.** Influence of EPs on the bioavailability of PVC-A MNPs. **D.** Influence of EPs on the bioavailability of PVC-I MNPs. mRNA expression log_2 fold changes for EP-PVC-A and EP-PVC-I MNPs were calculated compared to the expression levels observed in cells exposed to PVC-A and PVC-I, respectively.

References

(1) I. van der Veen, L. M. v. M., M.J.M. van Velzen, Q.R. Groenewoud, H.A. Leslie. *Plastic Particles in Livestock Feed, Milk, Meat and Blood A Pilot Study*; Vrije Universiteit Amsterdam, Plastic Soup Foundation, 2022. https://www.plasticsoupfoundation.org/rapporten/Final-Report-pilot-study-plastic-particles-in-livestock-feed-milk-meat-and-blood.pdf. Dessì, C.; Okoffo, E. D.; O'Brien, J. W.; Gallen, M.; Samanipour, S.; Kaserzon, S.; Rauert, C.; Wang, X.; Thomas, K. V. Plastics contamination of store-bought rice. *Journal of Hazardous Materials* **2021**, *416*, 125778. DOI: 10.1016/j.jhazmat.2021.125778. Ribeiro, F.; Okoffo, E. D.; O'Brien, J. W.; Fraissinet-Tachet, S.; O'Brien, S.; Gallen, M.; Samanipour, S.; Kaserzon, S.; Mueller, J. F.; Galloway, T.; Thomas, K. V. Quantitative Analysis of Selected Plastics in High-Commercial-Value Australian Seafood by Pyrolysis Gas Chromatography Mass Spectrometry. *Environmental Science & Technology* **2020**, *54* (15), 9408-9417. DOI: 10.1021/acs.est.0c02337.

(2) Gomiero, A.; Øysæd, K. B.; Palmas, L.; Skogerbø, G. Application of GCMS-pyrolysis to estimate the levels of microplastics in a drinking water supply system. *Journal of Hazardous Materials* **2021**, *416*, 125708. DOI: 10.1016/j.jhazmat.2021.125708. Xu, Y.; Ou, Q.; Jiao, M.; Liu, G.; van der Hoek, J. P. Identification and Quantification of Nanoplastics in Surface Water and Groundwater by Pyrolysis Gas Chromatography–Mass Spectrometry. *Environmental Science & Technology* **2022**, *56* (8), 4988-4997. DOI: 10.1021/acs.est.1c07377.

(3) Majumder, S.; Bazina, L.; DeLoid, G.; Garcia, A. G.; Zuverza-Mena, N.; Konkol, J.; Tsilomelekis, G.; Verzi, M.; Zhu, H.; White, J. C.; Demokritou, P. Impact of UV Aging on the Toxicity and Bioavailability of Inductively Coupled Plasma Mass Spectrometry (ICP-MS)-Traceable Core–Shell Polystyrene Nanoplastics in an In Vitro Triculture Small Intestinal Epithelium Model. In *Toxics*, 2025; Vol. 13, p 939.

(4) Tchounwou, P. B.; Yedjou, C. G.; Patlolla, A. K.; Sutton, D. J. Heavy metal toxicity and the environment. *Exp Suppl* **2012**, *101*, 133-164. DOI: 10.1007/978-3-7643-8340-4_6 From NLM.

(5) Wang, P.; Shi, Y.-Z.; Guan, Q. The Microplastic–PFAS Nexus: From Co-Occurrence to Combined Toxicity in Aquatic Environments. *Toxics* **2025**, *13* (12), 1041.

(6) Peña, A.; Rodríguez-Liébana, J. A.; Delgado-Moreno, L. Interactions of Microplastics with Pesticides in Soils and Their Ecotoxicological Implications. *Agronomy* **2023**, *13* (3), 701.

(7) Shankar, S.; Shanker, U.; Shikha. Arsenic contamination of groundwater: a review of sources, prevalence, health risks, and strategies for mitigation. *The scientific world journal* **2014**, *2014* (1), 304524. Georgaki, M.-N.; Charalambous, M. Toxic chromium in water and the effects on the human body: a systematic review. *Journal of Water and Health* **2022**, *21* (2), 205-223. DOI: 10.2166/wh.2022.214 (acccessed 7/2/2025). Jarvis, P.; Fawell, J. Lead in drinking water – An ongoing public health concern? *Current Opinion in Environmental Science & Health* **2021**, *20*, 100239. DOI: 10.1016/j.coesh.2021.100239.

(8) Angioni, A.; Dedola, F.; Garau, V. L.; Schirra, M.; Caboni, P. Fate of iprovalicarb, indoxacarb, and boscalid residues in grapes and wine by GC-ITMS analysis. *J Agric Food Chem* **2011**, *59* (12), 6806-6812. DOI: 10.1021/jf2011672 From NLM. Wee, S. Y.; Aris, A. Z. Revisiting the "forever chemicals", PFOA and PFOS exposure in drinking water. *npj Clean Water* **2023**, *6* (1), 57. DOI: 10.1038/s41545-023-00274-6.

(9) DeLoid, M. G. W., Y.; Kapronezai, K.; Lorente, L.R.; Zhang, R.; Pyrgiotakis, G.; Konduru, N.V.; Ericsson, M.; White, J.C.; De La Torre-Roche, R.; Xiao, H.; McClements, J.D.; Demokritou, P. An integrated methodology for assessing the impact of food matrix and gastrointestinal effects on the biokinetics and cellular toxicity of ingested engineered nanomaterials. *Particle and Fibre Toxicology* **2017**, *14* (1), 40. DOI: 10.1186/s12989-017-0221-5. DeLoid, G. M.; Yang, Z.; Bazina, L.; Kharaghani, D.; Sadrieh, F.; Demokritou, P. Mechanisms of ingested polystyrene micro-nanoplastics (MNPs)

uptake and translocation in an in vitro tri-culture small intestinal epithelium. *Journal of Hazardous Materials* **2024**, *473*, 134706. DOI: 10.1016/j.jhazmat.2024.134706.

(10) Kharaghani, D.; DeLoid, G. M.; Bui, T. H.; Zuverza-Mena, N.; Tamez, C.; Musante, C.; White, J. C.; Demokritou, P. Ingested Polystyrene Micro-Nanoplastics Increase the Absorption of Co-Ingested Arsenic and Boscalid in an In Vitro Triculture Small Intestinal Epithelium Model. In *Microplastics*, 2025; Vol. 4.

(11) Stock, V. B., L.; Lisicki, E.; Block, R.; Cara-Carmona, J.; Pack, L.K.; Selb, R.; Lichtenstein, D.; Voss, L.; Henderson, C.J.; Zabinsky, E.; Sieg, H.; Braeuning, A.; Lampen, A. Uptake and effects of orally ingested polystyrene microplastic particles in vitro and in vivo. *Archives of Toxicology* **2019**, *93* (7), 1817-1833. DOI: 10.1007/s00204-019-02478-7.

(12) Kharaghani, D.; DeLoid, G. M.; He, P.; Swenor, B.; Bui, T. H.; Zuverza-Mena, N.; Tamez, C.; Musante, C.; Verzi, M.; White, J. C.; Demokritou, P. Toxicity and absorption of polystyrene micro-nanoplastics in healthy and Crohn's disease human duodenum-chip models. *Journal of Hazardous Materials* **2025**, *490*, 137714. DOI: 10.1016/j.jhazmat.2025.137714.

(13) Bray, N. L.; Pimentel, H.; Melsted, P.; Pachter, L. Near-optimal probabilistic RNA-seq quantification. *Nature Biotechnology* **2016**, *34* (5), 525-527. DOI: 10.1038/nbt.3519.

(14) Love, M. I.; Huber, W.; Anders, S. Moderated estimation of fold change and dispersion for RNA-seq data with DESeq2. *Genome biology* **2014**, *15*, 1-21.

(15) Das, M. C., L.; Singh D.; Majumder, S.; Bazina, L.; Vaze, N.; Trivanovic, U.; DeLoid, G.; Zuverza-Mena, N.; Kaur, M.; Konkol, J.; Tittikpina, K.N.; Tsilomelekis, G.; Sadik, O.; White, J.C.; Demokritou, P. Development and characterization of reference environmentally relevant micro-nano-plastics for risk assessment studies. *NanoImpact* **2025**, *38*, 100567. DOI: 10.1016/j.impact.2025.100567.
